# Bacteria–organelle communication in physiology and disease

**DOI:** 10.1083/jcb.202310134

**Published:** 2024-05-15

**Authors:** Yi-Tang Lee, Mumine Senturk, Youchen Guan, Meng C. Wang

**Affiliations:** 1 Waisman Center, University of Wisconsin, Madison, WI, USA; 2 Huffington Center on Aging, Baylor College of Medicine, Houston, TX, USA; 3 Integrative Program of Molecular and Biochemical Sciences, Baylor College of Medicine, Houston, TX, USA; 4 Howard Hughes Medical Institute, Baylor College of Medicine, Houston, TX, USA; 5 Department of Molecular and Cellular Biology, Baylor College of Medicine, Houston, TX, USA; 6 Janelia Research Campus, Howard Hughes Medical Institute, Ashburn, VA, USA

## Abstract

Bacteria, omnipresent in our environment and coexisting within our body, exert dual beneficial and pathogenic influences. These microorganisms engage in intricate interactions with the human body, impacting both human health and disease. Simultaneously, certain organelles within our cells share an evolutionary relationship with bacteria, particularly mitochondria, best known for their energy production role and their dynamic interaction with each other and other organelles. In recent years, communication between bacteria and mitochondria has emerged as a new mechanism for regulating the host’s physiology and pathology. In this review, we delve into the dynamic communications between bacteria and host mitochondria, shedding light on their collaborative regulation of host immune response, metabolism, aging, and longevity. Additionally, we discuss bacterial interactions with other organelles, including chloroplasts, lysosomes, and the endoplasmic reticulum (ER).

## Teaser

Meng Wang and colleagues review the intricate communication between bacteria and host organelles and the impact of such communication on immune response, aging, and longevity.

## Introduction

In a world seemingly dominated by our species, boasting an impressive global headcount of 8 billion, a closer examination reveals an astonishing microbial superpower that overshadows our human presence: bacteria. More than a nonillion (10^30^) of these microorganisms inhabit our planet, encompassing millions of distinct species (Flemming and Wuertz, 2019). Some of them are disease-causing pathogens, while others are essential contributors to human health. Remarkably, a substantial portion of these microorganisms reside as our commensal companions. It is estimated that around 38 trillion bacteria thrive within or upon the average 70-kg male human, surpassing the total count of human cells in the body (∼30 trillion) (Sender et al., 2016). An imbalance among commensal bacteria has been linked with pathological alterations (Cho and Blaser, 2012). For example, alterations in the gut microbiome have been associated with a spectrum of ailments, encompassing neurodegenerative diseases, cardiovascular diseases, gastrointestinal diseases, cancers, and more (Cullin et al., 2021; Fang et al., 2020; Jie et al., 2017; Trakman et al., 2022). The gut microbiome’s role in modulating aging and longevity further underscores their impact on human health (O’Toole and Jeffery, 2015; Wilmanski et al., 2021).

It is interesting to note that the symbiotic interaction between humans and bacteria extends from the macroscopic to the microscopic scale. Eukaryotic cells, including human cells, harbor a variety of organelles, such as mitochondria, lysosomes, the endoplasmic reticulum (ER), and the nucleus, each with distinctive activities. Mitochondria are the powerhouse of eukaryotic cells, which provide essential energy to support all cellular processes. These organelles also dynamically interact with each other and with other organelles, playing crucial roles in cellular signaling. The prevailing hypothesis suggests that mitochondria originated through an endosymbiotic relationship between bacteria and amitochondriate eukaryote or methanogenic archaea, which eventually led to a blooming diversity of eukaryotic species (Gray et al., 1999; López-García and Moreira, 2020; Martin et al., 2015). Multiple phylogenetic studies suggest a close kinship between mitochondria and the α-proteobacteria Rickettsiales (Andersson et al., 1998, 2003; Fitzpatrick et al., 2006), although recent findings have cast a discerning light on this proposition (Geiger et al., 2023; Martijn et al., 2018; Roger et al., 2017).

Cumulative evidence reveals that pathogens can target mitochondria in the host cell to facilitate their infection, while host cells employ these very organelles in their defense against pathogenic invaders. Emerging studies also uncover that the microbiome influences host physiology through communicating with mitochondria. In this review, we summarize the communication between bacteria and mitochondria, exploring their collaborative role in regulating host disease, aging, and longevity. Furthermore, this review also provides insights into the communications of bacteria with other organelles in addition to mitochondria. We define bacteria–organelle communication as the process through which bacteria influence organellar architecture and/or activities, and organelle/bacteria communication as the process through which organelles/bacteria interact with each other via physical contacts and exchanging chemical molecules, such as ions and metabolites.

### Origin of mitochondria

The prevailing mitochondrial origin theory, known as the endosymbiotic theory (Gray and Doolittle, 1982; Roger et al., 2017; Sagan, 1967; Schwartz and Dayhoff, 1978; Yang et al., 1985), suggests that mitochondria are descendants of free-living bacterial ancestors via the process of endosymbiosis. The Serial Endosymbiosis Theory, a version of endosymbiotic theory proposed by Lynn Margulis (Gray et al., 1999; Sagan, 1967), suggests that the aerobic mitochondrial endosymbiont, enabling oxygen detoxification and energy production, conferred advantages to the anaerobic host cell. This process eventually led to the evolution of specialized intracellular organelles. An alternative perspective within the endosymbiotic theory, the Hydrogen Hypothesis (Martin and Müller, 1998), shifts the focus away from the primacy of endosymbiosis for energy production via oxidative phosphorylation. Instead, it emphasizes that the association between the host cell and the endosymbiont is primarily driven by the exchange of hydrogen, with the eubacterial symbiont generating hydrogen for use by the hydrogen-dependent host cell. The endosymbiotic theory is supported by the striking similarities between mitochondria and bacteria, including the presence of a double membrane structure, circular double-stranded DNA, tRNA, ribosomes, and enzymes, as well as the ability to undergo replication independently (Pallen, 2011).

Phylogenetic analyses have bolstered the case of mitochondria’s bacterial origins (Gray et al., 1999) and illustrated that mitochondria have an alphaproteobacterial ancestry. When considering the closest eubacterial relatives in the alphaproteobacterial taxa, most studies report parasitic Rickettsiales (Andersson et al., 1998, 2003; Fitzpatrick et al., 2006), whereas other studies suggest free-living Rhizobiales, Rhodobacterales, and Rhodospirillales (Atteia et al., 2009; Esser et al., 2004). The variation in the placement of mitochondria is likely due to different phylogenetic approaches that are used in these studies (Roger et al., 2017), as well as the high phylogenetic divergence within Alphaproteobacteria, strong statistical noise and artefactual signals, and limitation of taxonomy (Fan et al., 2020).

### Bacteria–bacteria communication

Bacteria and mitochondria not only share evolutionary origins but also exhibit similar communication within their community. To understand how bacterial communities influence the homeostasis of the organelles in the host cells, especially the mitochondria, it is essential to explore how bacteria communicate with each other.

**Figure 1.**
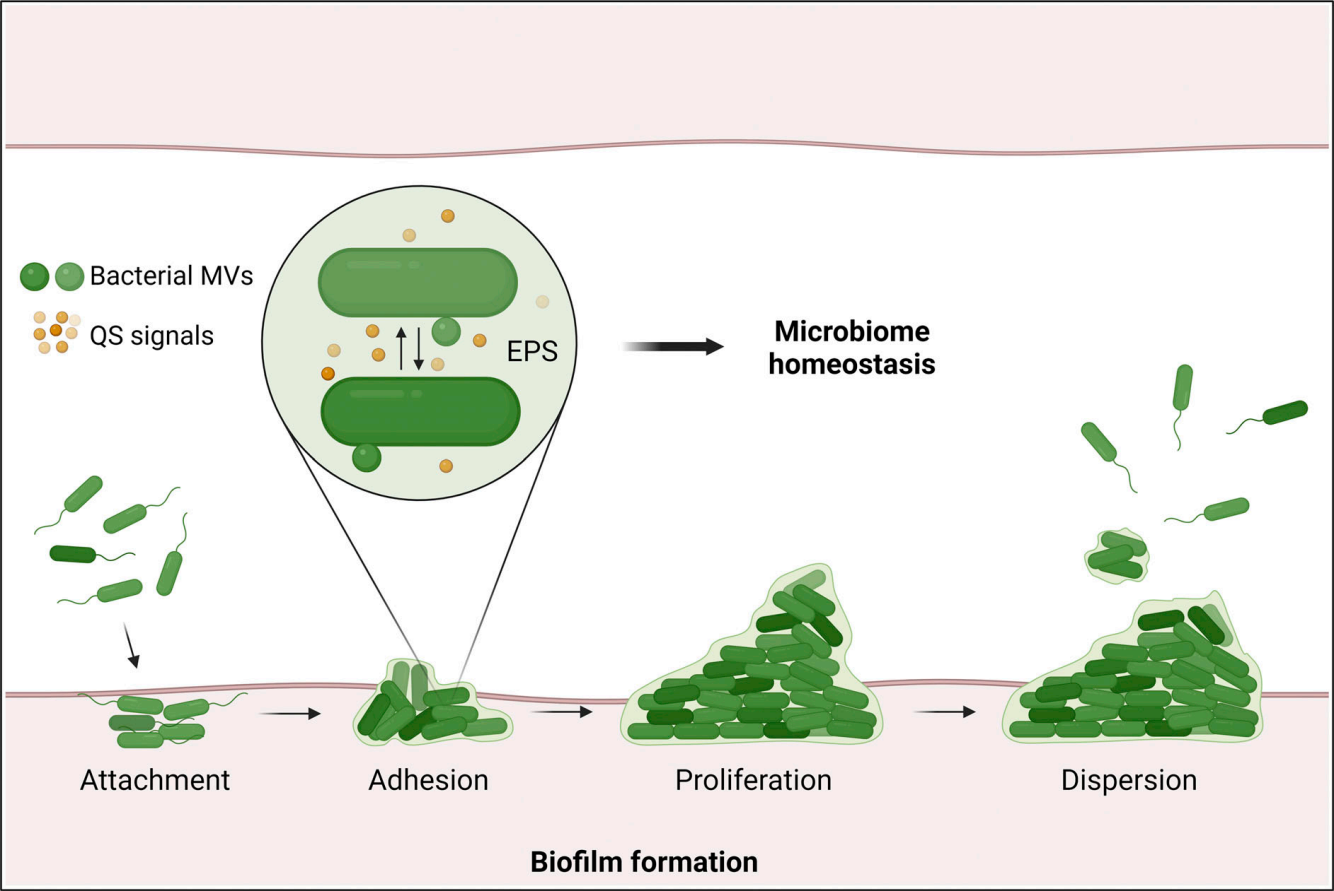
**Bacteria exhibit communal behavior through various communication strategies.** Biofilms serve as a form of communal existence, where bacteria aggregate, forming extracellular polymeric substance (EPS) matrices that offer protection, nutrient absorption, habitat diversity, and social interactions. Bacteria can also employ quorum sensing (QS), releasing signals to coordinate gene expression based on population density. Bacterial membrane vesicles (MVs) facilitate the transfer of diverse cargos, including hydrophobic signals involved in QS.

Given the small size of bacteria, it is not surprising that these microorganisms always operate as a community through their intricate communication strategies. One such form of communal existence is the biofilm, which offers various benefits to the bacteria residing within it, compared with free-living bacteria. These benefits include protection against adverse conditions, facilitation of nutrient absorption, creation of localized gradients for habitat diversity, and promotion of social interactions (Flemming et al., 2016). Within the biofilm, bacteria attach to each other and aggregate into clusters, which involves the production of extracellular polymeric substance (EPS) and the formation of the EPS matrix (Fig. 1). Bacteria residing in biofilms can contribute to infectious diseases, exacerbate chronic infections, and incite infections via medical devices by evading host immune responses and antibiotic treatments (Vestby et al., 2020).

Beyond direct cell-to-cell contact, many bacteria can also employ chemical signaling molecules, such as autoinducers, to communicate with each other, a process known as quorum sensing (QS). During QS, bacteria release autoinducers to the surrounding environment, where their external concentration corresponds to the population density (Fig. 1). As the autoinducer accumulates and reaches a critical stimulatory concentration, bacteria start to trigger specific gene expression programs that regulate community behavior (Keller and Surette, 2006; Waters and Bassler, 2005). Interestingly, QS signals in the host gut microbiome not only help inhibit potential pathogens (Piewngam et al., 2018; Wu and Luo, 2021) but also modulate host signaling transduction (Hughes and Sperandio, 2008).

Moreover, bacteria communication is facilitated by outer membrane vesicles (OMVs) originating from the outer surface of bacteria. OMVs serve as messengers, transferring various cargo, such as proteins, metabolites, nucleic acids, and toxins, between bacterial cells (Caruana and Walper, 2020). In particular, OMVs are important for the transport of hydrophobic signals that are not able to freely diffuse out of the cell to induce QS (Toyofuku, 2019; Toyofuku et al., 2017). During infection, OMVs can be internalized by host cells through the endocytosis process, releasing their cargo, such as virulence factors. This process thereby regulates molecular pathways within the host cells and functions of organelles including lysosomes, eventually causing cell damage and suppressing the immune system (O’Donoghue and Krachler, 2016). Understanding these bacteria–bacteria communication cues helps to unravel the complex interactions between bacteria and their hosts.

### Mitochondria in response to pathogens

The interactions between pathogenic bacteria and their hosts play a central role in immune response. Bacterial effectors, which are proteins secreted by pathogenic bacteria into the host’s cell, target mitochondria to modulate cellular death. Pathogenic bacteria also shape the host’s mitochondrial network to facilitate their infection. Importantly, not only do pathogenic bacteria modulate host mitochondria for their own benefit, but the host also utilizes mitochondria for its own defense against bacterial infection.

#### Bacterial effectors target mitochondria to regulate apoptosis

Bacteria developed diverse secretion systems to transport proteins across their cellular membrane. These secretion systems vary in complexity and function. Type I, III, IV, and VI secretion systems are able to transport effectors across both inner and outer membranes, and even directly into the host cell in the case of type III, IV, and VI secretion systems. On the other hand, type II, V, IX, and XI secretion systems require exportation pathways to transport the effectors into the periplasm before further secretion (Arechaga and Cascales, 2022; Green and Mecsas, 2016).

The effectors of the bacterial type III secretion system (T3SS), which has been reported to be mainly utilized by gram-negative bacteria, have been found to activate or suppress the apoptosis machinery by affecting mitochondria (Nandi et al., 2021). Upon infection, the enteropathogenic *E. coli* secretes numerous effectors, including the T3SS-dependent *E. coli* secreted proteins F (EspF), mitochondrial-associated protein (Map), and *E. coli*-secreted protein Z (EspZ) (Serapio-Palacios and Finlay, 2020). Both EspF and Map possess mitochondria-targeting sequences (MTS) in their N-terminus, allowing their entry into the host mitochondria (Kenny and Jepson, 2000; Nougayrède and Donnenberg, 2004), which results in disrupted mitochondrial membrane potential and apoptosis activation (Ma et al., 2006; Nagai et al., 2005; Ramachandran et al., 2020). On the contrary, EspZ has been found to elicit an anti-apoptotic effect (Shames et al., 2010). How these effectors coordinate with each other to promote enteropathogenic *E. coli* infection remains to be explored. Moreover, the T3SS-dependent effector, invasion plasmid antigen D (IpaD), in the enteroinvasive bacteria *Shigella flexneri* (*S. flexneri*) was reported to disrupt mitochondrial membrane potential and induce apoptosis in macrophages, although its mitochondrial localization has not been confirmed (Arizmendi et al., 2016).

For the bacterial type IV secretion system (T4SS), although it is largely found in gram-negative bacteria, it has also been identified in gram-positive bacteria and archaea (Sheedlo et al., 2022). Ehrlichiosis is a tick-borne disease that is caused by infection of the obligate intracellular bacteria *Ehrlichia chaffeensis* (*E. chaffeensis*). *E. chaffeensis* secretes the T4SS-dependent effector Ehrlichia translocated factor-1 (Etf-1), which targets mitochondria via its MTS and has been reported to be anti-apoptotic (Liu et al., 2012). Interestingly, expressing the nanobody that targets Etf-1 blocks its mitochondrial localization and abrogates its inhibition of etoposide-induced apoptosis in non-human primate cell lines infected with *E. chaffeensis* (Zhang et al., 2021).

**Figure 2.**
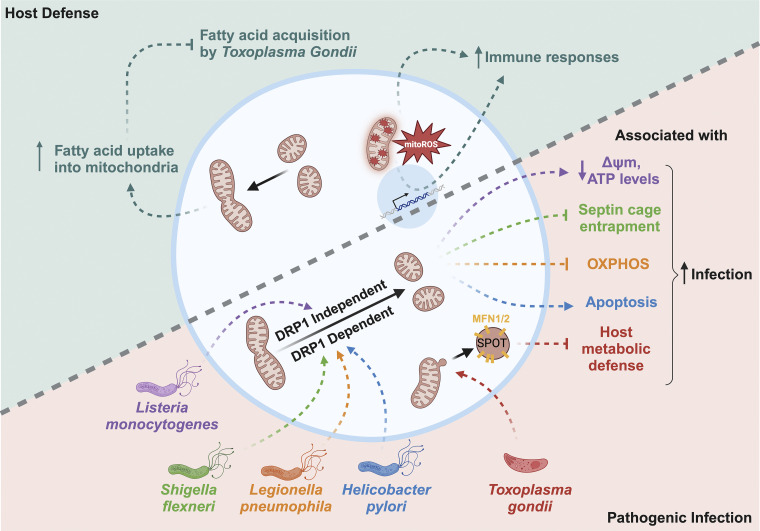
**Pathogens and mitochondria interact during infection.** *Shigella flexneri*, *Legionella pneumophila*, and *Helicobacter pylori* drive mitochondrial fragmentation in a DRP1-dependent manner, while *Listeria monocytogenes* do so via a DRP1-independent mechanism, facilitating pathogenic infection through various pathways. *Toxoplasma gondii* remodels the mitochondria outer membrane into SPOT structure that contains mitochondrial fusion machinery such as MFN1/2, blocking the host metabolic defense and favoring infection. On the other hand, mitochondria serve as the host intracellular response center to defend against bacterial infection via promoting metabolic reprogramming and inducing mitoROS-mediated immune response.

There are also non-T3SS and -T4SS effectors that target mitochondria to modulate apoptosis. For example, infection of *Helicobacter pylori (H. pylori)* causes apoptosis in gastric epithelial cells, which is mediated by the secreted vacuolating cytotoxin A (VacA). After endocytosis into the epithelial cell, VacA is imported into mitochondria at the endosome–mitochondria juxtaposition and then inserted into the inner mitochondrial membrane to form an anion-selective pore. The permeabilization of the mitochondrial outer membrane disrupts membrane potential, resulting in the release of cytochrome c and the induction of apoptosis (Fig. 2) (Czajkowsky et al., 1999; Domańska et al., 2010; Galmiche and Rassow, 2010; Galmiche et al., 2000; Jain et al., 2011). It was also observed that upon incubation with VacA, Dynamin-related protein 1 (DRP1) is recruited to the mitochondria, leading to mitochondrial fission. Another example is the intracellular bacterium *Legionella pneumophila* (*L. pneumophila*) effector Lpg1137, which inhibits apoptosis through cleavage of syntaxin 17 (Stx17), a protein located at the mitochondria–ER contact site (Arasaki et al., 2017; Hamasaki et al., 2013). Mitochondria-associated ER membranes (MAMs) in the mitochondria–ER contact site have been implicated in the regulation of various crucial cellular activities, including apoptosis, calcium homeostasis, mitochondrial dynamics, and autophagosome formation. Consequently, MAMs are believed to be prime targets for pathogenic bacteria (Escoll et al., 2017a; Raturi and Simmen, 2013). It is known that Stx17 interacts with Drp1 at MAMs to promote mitochondrial fission, an early event often observed upon apoptotic cell death (Arasaki et al., 2015; Suen et al., 2008). Through degrading Stx17, Lpg1137 abrogates the Stx17-Drp1 interaction and blocks apoptosis, which could facilitate the continual replication of *L. pneumophila* (Abu-Zant et al., 2005; Arasaki et al., 2017).

#### Pathogenic bacteria tuning mitochondrial dynamics

The mitochondrial network maintains a dynamic equilibrium between fragmented and tubular morphologies, a balance crucially intertwined with mitochondrial metabolic activities and their interactions with other organelles (Box 1, mitochondrial fission-fusion dynamics). Manipulation of mitochondrial dynamics by pathogenic bacteria not only influences cell death but also helps them evade degradation and obtain energy for replication.

Box 1 Mitochondrial fission–fusion dynamics

Mitochondria are highly dynamic organelles that constantly adapt their morphology and cellular distribution in response to stress or metabolic changes, displaying high heterogeneity at multiple levels. For instance, studies showed that severe stress such as prolonged nutrient deprivation and electron transport chain (ETC) inhibition leads to fission while mild stressors such as inhibition of RNA transcription or protein translation, UV irradiation, and moderate starvation cause fusion (Tondera et al., 2009; Toyama et al., 2016; Zemirli et al., 2018). Mitochondrial fission–fusion processes are critical for mitochondria to maintain their appropriate size, shape, and function, which is regulated by a complex network of proteins. Three dynamin-like GTPases, dynamin-related protein 1 (DRP1), mitofusins (MFN1/2), and optic atrophy protein 1 (OPA1), are key regulators of mitochondrial fission and fusion, which are activated and inactivated by posttranslational modifications and can be modulated by interactions with various regulatory proteins. Fission is initiated by DRP1 recruitment to mitochondria by several adaptor proteins such as Fission 1 (FIS1), followed by assembly into a ring-like structure that constricts the organelle and eventually divides it (Chan, 2006; Westermann, 2010). Fusion, on the other hand, is facilitated by MFN1/2 and OPA1 which mediate the tethering and fusion of two adjacent mitochondria, leading to the creation of highly interconnected networks (Chan, 2006; Westermann, 2010).

Although fission and fusion exert distinct effects on mitochondrial functions, they both play a vital role in cell and tissue homeostasis. Mitochondrial fusion enables the exchange of mitochondrial contents and DNA between mitochondria and disruption of mitochondrial fusion causes mitochondrial dysfunction (Adebayo et al., 2021; Chen et al., 2005; Yang et al., 2015). In addition, mitochondrial fusion contributes to enhancing the rate of lipid consumption by facilitating the distribution of fatty acids from lipid droplets among the mitochondrial network and consequently beta-oxidation of these fatty acids by mitochondria (Rambold et al., 2015). Also, studies in mice showed that obesity increases mitochondria fragmentation, and adipose tissue expression of Drp1 displays a positive correlation with obesity and insulin resistance (Xia et al., 2024).

On the other hand, mitochondrial fission can enable both the selective removal of damaged organelles through mitophagy and mitochondrial biogenesis (Burman et al., 2017; Kleele et al., 2021). Interestingly, mitochondrial fission and fusion dynamics also control the positioning of mitochondria within the cell to accommodate cellular metabolic needs (Han et al., 2012; Lee et al., 2023; Wakai et al., 2014) and their physical/functional crosstalk with other organelles.

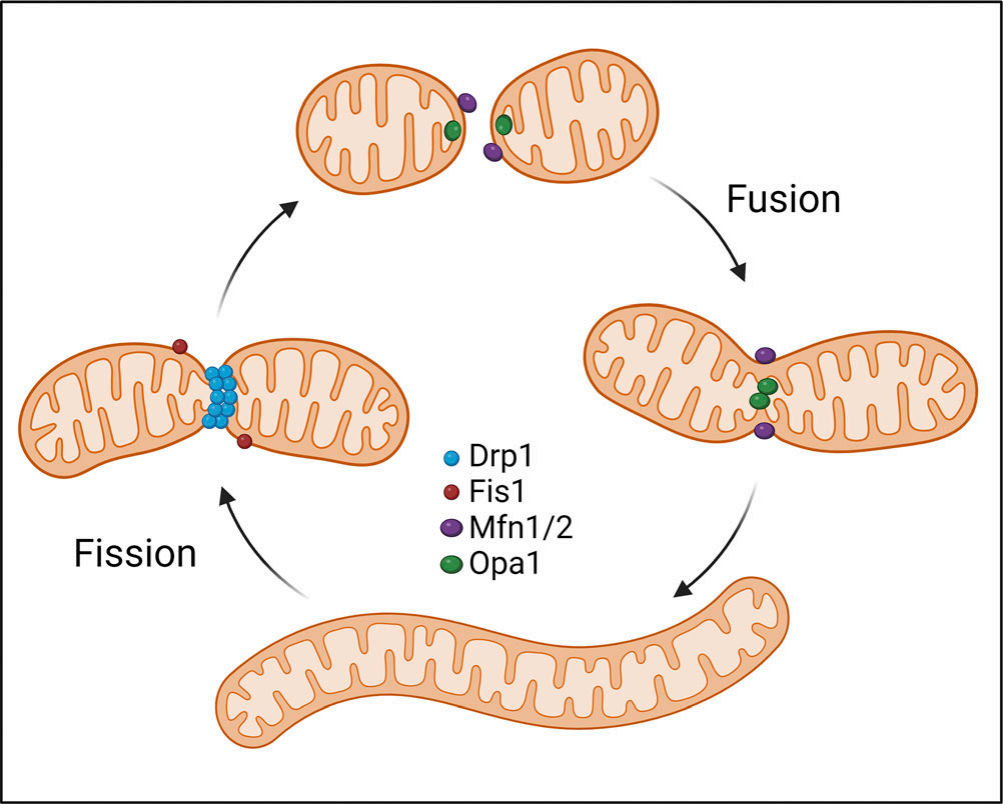

*S. flexneri* promotes mitochondrial fragmentation in a DRP1-dependent manner (Lum and Morona, 2014). During infection, the septin protein in the host forms a cage to trap *S. flexneri* (Mostowy et al., 2010), which is then targeted to the autophagosome for degradation (Mostowy et al., 2011). It was shown that effective trapping of *S. flexneri* by the septin cage relies on a non-fragmented mitochondrial network. Thus, *S. flexneri*-induced mitochondrial fragmentation may serve to protect the pathogen from entrapment (Fig. 2) (Sirianni et al., 2016). *H. pylori* effector VacA also promotes mitochondrial fission through a DRP1-dependent manner, leading to cell death (Fig. 2) (Jain et al., 2011). On the other hand, while *L. pneumophila* inhibits apoptosis, its infection nonetheless promotes mitochondrial fragmentation. This process is mediated by the secreted T4SS effector MitF, a Ran GTPase activator, that induces DRP1 accumulation to drive fission (Escoll et al., 2017b). The fragmented mitochondria in macrophages impair mitochondrial oxidative phosphorylation, and together with T4SS-independent induction of glycolysis, a Warburg-like effect is provoked. This metabolic reprogramming in host cells favors bacterial replication (Fig. 2) (Escoll et al., 2017b). In the case of *Listeria monocytogenes* (*L. monocytogenes*), it induces mitochondrial fragmentation through a DRP1-independent mechanism. Prior to bacterial entry, listeriolysin O secreted by *L. monocytogenes* forms calcium-permeable pores on the plasma membrane and causes mitochondrial fragmentation accompanied by a reduction in mitochondrial membrane potential and intracellular ATP levels (Fig. 2) (Repp et al., 2002; Stavru et al., 2011). Interestingly, this listeriolysin O–induced fragmentation of the host mitochondrial network does not require Drp1 or Opa1 but takes place at the mitochondria–ER contact site (Stavru et al., 2013).

Despite most reported cases showing fragmentation of mitochondria upon bacterial infection, tubulation of the host mitochondrial network was observed in primary cells infected by the obligate intracellular bacterium *Chlamydia trachomatis* (*C. trachomatis*) during the early phase (Chowdhury et al., 2017). This process is mediated by the downregulation of DRP1 and is associated with increased mitochondrial oxygen consumption and intracellular ATP content (Kurihara et al., 2019). However, during prolonged infection with *C. trachomatis*, mitochondrial fragmentation begins to take place (Kurihara et al., 2019), suggesting that early-phase mitochondrial tubulation may allow the pathogen to gain access to more ATP molecules, further develop, and establish the infection. Moreover, the impact of pathogenic bacteria on mitochondria is not limited to mitochondrial fission-fusion dynamics. During *Vibrio cholerae* (*V. cholerae*) infection, perinuclear clustering of mitochondria is inhibited, suppressing innate immune responses in mammalian cells (Suzuki et al., 2014). This inhibition is mediated by VopE, a T3SS effector, which binds to mitochondrial Rho GTPases Miro1 and Miro2 to interfere with their modulation of mitochondrial trafficking (Suzuki et al., 2014).

#### Mitochondrial responses to bacterial infection

Mitochondrial reactive oxygen species (mitoROS) is central to the cellular immune response to pathogens (Fig. 2) (Pinegin et al., 2018). Lipopolysaccharides (LPS), lipoproteins, and other toxins released by bacteria are known as pathogen-associated molecular patterns (PAMPs), which are recognized by the host pattern recognition receptors (PRRs), including Toll-like receptors (TLRs) and Nod-like receptors (NLRs) (Tiku et al., 2020; West et al., 2011). In macrophages, the activation of TLR1, 2, and 4 induces mitoROS production and recruits mitochondria toward phagosomes (West et al., 2011). NLRX1, a member of the NLR family, is targeted to the mitochondrial matrix, and its activation enhances mitoROS production as well (Arnoult et al., 2009; Tattoli et al., 2008). The bactericidal effect of mitoROS occurs at multiple levels. mitoROS can directly exert oxidative damage to the pathogen (Abuaita et al., 2018; Geng et al., 2015; West et al., 2011). It can also activate NADPH oxidases in the phagosome to further generate ROS for bacterial killing (Kröller-Schön et al., 2014). In macrophages infected by methicillin-resistant *Staphylococcus aureus*, it was reported that mitoROS is delivered to phagosomes via mitochondria-derived vesicles upon infection-induced ER stress (Abuaita et al., 2018). Furthermore, mitoROS is a crucial signaling molecule that activates transcriptional factors such as NFkB (Chen et al., 2011; Gloire et al., 2006; Zinovkin et al., 2014), and members of the NLR family, NLRP3 and NLRC4, to upregulate proinflammatory cytokines and initiate the assembly of inflammasomes, respectively (Jabir et al., 2015; Shimada et al., 2012).

Additionally, metabolic reprogramming orchestrated by mitochondria is crucial for different aspects of immune responses. A prominent example is that macrophages undergo a metabolic shift from oxidative phosphorylation to aerobic glycolysis upon encountering bacteria (Rosenberg et al., 2022), which promotes proinflammatory responses. In LPS-activated macrophages, TCA cycle enzymes isocitrate dehydrogenase 1 (IDH1) is transcriptionally repressed (De Souza et al., 2019), and both IDH1 and aconitase are functionally inhibited (Bailey et al., 2019; Palmieri et al., 2020), leading to the accumulation of citrate and cis-aconitate. The cis-aconitate is further converted to itaconate by the mitochondrial enzyme cis-aconitate decarboxylase, and itaconate exerts its bactericidal activity by inhibiting bacterial isocitrate lyase and propionyl-CoA carboxylase, resulting in disruptions of the glyoxylate cycle and acetate assimilation that are important for bacterial survival (Berg et al., 2002; Peace and O’Neill, 2022). Interestingly, enzymes that facilitate the degradation of itaconate into pyruvate and acetyl-CoA are found in numerous pathogenic bacteria, and the genes encoding these enzymes have been shown to be essential for the survivability of some pathogens inside the macrophage (Sasikaran et al., 2014).

#### Host mitochondrial association by *Toxoplasma gondii*

In addition to bacteria, another pathogen that exhibits a close association with the host’s mitochondria is the intracellular parasite *Toxoplasma gondii* (*T. gondii*). It is known that upon infection, *T. gondii* resides in vacuoles and tethers host mitochondria to vacuoles via the secreted *Toxoplasma gondii* mitochondrial association factor 1 (TgMAF1) protein (Pernas et al., 2014; Sinai et al., 1997). Although the detailed mechanism remains to be explored, *T. gondii* acquires fatty acids for growth and proliferation by promoting lipid droplet incorporation into the vacuoles and inducing lipophagy in the host (Nolan et al., 2017; Pernas et al., 2018). Simultaneously, the mitochondrial network surrounding the vacuoles fuses together and becomes tubular in the early phase of the infection (Pernas et al., 2014), leading to increased fatty acid uptake into mitochondria, which competes with *T. gondii* for nutrients. When mitochondrial fusion is lacking, *T. gondii* growth and proliferation are enhanced (Pernas et al., 2018). Interestingly, the outer mitochondrial membrane (OMM) translocase TOM70 (translocase of the outer membrane 70) and the mitochondria-specific chaperon HSPA9 have been identified as the host binding partners of TgMAF1, which are required for mitochondrial tethering to vacuoles upon *T. gondii* infection (Blank et al., 2021).

More recently, the study of *T. gondii* has revealed its infection as a natural stressor that triggers the remodeling of the outer mitochondrial membrane (OMM) (Li et al., 2022). Upon infection, structures known as SPOTs (structures positive for OMM) emerge within the host cell (Fig. 2). These structures exclusively contain OMM proteins but lack proteins located in the mitochondrial matrix or the inner mitochondrial membrane. The formation of SPOTs requires TOM70, which mediates the binding between TgMAF1 and another OMM translocase SAM50 (sorting assembly machinery 50 kDa subunit). The shedding of SPOTs from the mitochondria results in the depletion of OMM proteins, including Mfn1 and Mfn2, which are required for mitochondrial fusion to compete for nutrients with the parasite. The loss of other OMM translocases required for mitochondrial biogenesis also diminishes the nutritional defense mediated by host mitochondria. Interestingly, without infection, the removal of SAM50 or the overexpression of alpha-helical OMM proteins is sufficient to induce SPOT formation, suggesting that *T. gondii* hijacks an intricate cellular response associated with OMM remodeling in the host cell for its own benefits (Li et al., 2022).

### Bacteria–mitochondria communication in aging and longevity

#### Mitochondria communication in aging

Like their prokaryotic relatives, mitochondria also dynamically communicate with each other, characterized by fusion, fission, and trafficking (Box 1), enabling them to adapt to the cell’s changing demands, ensuring optimal function and response to environmental cues. During aging, mitochondrial dynamics undergo changes, leading to a decline in the efficiency and integrity of mitochondria and ultimately contributing to the development of various age-related diseases (Liu et al., 2020; Srivastava, 2017). In particular, impaired mitochondrial dynamics caused by mitochondrial fission–fusion defects are well documented in neurodegenerative diseases such as Alzheimer’s disease (AD), Parkinson’s disease (PD), Huntington’s disease, Charcot-Marie-Tooth type 2A disease, and dominant optic atrophy (Alexander et al., 2000; Arduíno et al., 2012; Flannery and Trushina, 2019; Reddy, 2014; Su et al., 2010; Trimmer et al., 2000; Van Laar and Berman, 2009; Züchner et al., 2004).

Interestingly, manipulating mitochondrial fusion–fission regulators proves to be an effective way of altering lifespan and healthspan in model organisms (Table 1). Studies in the fruit fly *Drosophila melanogaster* showed that transient upregulation of Drp1 during midlife to promote mitochondrial fission prolongs healthspan, which is associated with increased mitochondrial membrane potential, youthful cristae ultrastructure, improved mitochondrial respiratory capacity, reduced levels of mitoROS, enhanced mitophagy, and proteostasis in aged flies (Rana et al., 2017). Reduction of mitochondrial fusion factors by overexpression of parkin E3 ubiquitin ligase in flies also improves mitochondrial activity and prolongs healthspan (Rana et al., 2013). Similarly, intestine-specific overexpression of *drp-1* extends *C. elegans’* lifespan (Han et al., 2017). In mice, Mfn2 deficiency in the skeletal muscle leads to disrupted mitochondrial dynamics and premature aging phenotypes, although the lifespan is not altered (Sebastián et al., 2016). On the other hand, in *C. elegans*, an increased mitochondrial elongation morphology, likely resulting from enhanced mitochondrial fusion, has been associated with several prolongevity pathways, including reduction of insulin/IGF-1 signaling, caloric restriction, mitochondrial ETC deficiency, mTOR inactivation, and AMPK activation (Chaudhari and Kipreos, 2017; Jacobi et al., 2015; Weir et al., 2017). However, the knockdown of *drp-1* or overexpression of *fzo-1* (MFN1/2 ortholog), both of which lead to an increase in mitochondrial fusion, does not increase *C. elegans’* lifespan (Weir et al., 2017). Thus, achieving a delicate balance between mitochondrial fusion and fission is critical for cellular health, while the connection between mitochondrial dynamics and longevity regulation is multifaceted, which may vary depending on the biological context and with tissue and age specificity.

**Table 1.** Alterations in mitochondrial dynamics affect lifespan in model organisms

Genetic manipulation	Effect on mitochondrial dynamics	Model organism	Lifespan	Reference
Transient upregulation of Drp1	Increased fission	*D. melanogaster*	Increase in lifespan	Rana et al., 2017
Parkin E3 overexpression	Increased fission	*D. melanogaster*	Increase in lifespan	Rana et al., 2017
Intestine-specific overexpression of drp-1	Increased fission	*C. elegans*	Increase in lifespan	Han et al., 2017
Mfn2 knockout in skeletal muscle	Impaired fusion, Swollen mitochondria upon aging	*Mice*	Premature aging, No change in lifespan	Sebastián et al., 2016
Knockdown of drp-1 or overexpression of fzo-1	Increased fusion	*C. elegans*	No change in lifespan	Weir et al., 2017

Furthermore, mitochondria communicate with other organelles via both membrane contact and molecular exchanges, which have also been linked with longevity regulation in model organisms. Mitochondrial retrograde signaling via mitochondrial unfolded protein response (mitoUPR) plays a crucial role in *C. elegans’* longevity regulation (reviewed in Lima et al., 2022). Improvements in mitochondrial functions by increased lysosomal acidity and lysosomal lipolysis have been shown to extend lifespan in yeast and *C. elegans*, respectively (Hughes and Gottschling, 2012; Ramachandran et al., 2019). Additionally, reducing ER–mitochondria Ca^2+^ exchange by deleting inositol 1,4,5-trisphosphate receptor (IP_3_R) improves both lifespan and healthspan in female mice (Ziegler et al., 2021), while in *C. elegans*, the gain-of-function mutation of IP_3_R increases lifespan (Burkewitz et al., 2020).

#### Bacterial metabolites act through mitochondria to regulate host aging

In addition to pathogenic bacteria, trillions of non-pathogenic bacteria in the microbiome coexist with eukaryotic organisms. There is a continuous exchange of nutrients, genetic materials, and metabolites between these commensal bacteria and their hosts. The metabolic inputs from bacteria play crucial roles in modulating the host’s physiological and pathological activities. Interestingly, emerging studies reveal mitochondria as key mediators of these bacteria-host metabolic interactions in regulating stress responses, metabolism, and aging.

Short-chain fatty acids (SCFAs) such as butyrate and acetate are among the best-studied metabolites produced by commensal bacteria through the fermentation of dietary fibers (LeBlanc et al., 2017). The levels of SCFAs alter during the aging process (Salazar et al., 2019), which has been implicated in neuroinflammation and various age-associated diseases (Bostick et al., 2022; Tran and Mohajeri, 2021). SCFAs’ supplementation has been shown to influence age-associated pathologies through their effects on mitochondria. For example, bacteria-derived acetate modulates mitochondrial membrane potential and electron transport chain (ETC) complex II activity in mouse microglia, contributing to microglial maturation and function during the steady state (Erny et al., 2021). Bacteria-derived acetate also modulates microglial phagocytosis and hippocampal amyloid-beta pathology in the 5xFAD mouse model of AD (Erny et al., 2021). In insulin-resistant obese mice, butyrate supplementation elevates mitochondrial fusion factors Mfn1, Mfn2, and Opa1 expression in the liver and increases mitochondrial functions in liver, brown adipose tissue, and skeletal muscle, leading to improved insulin sensitivity (Gao et al., 2009; Mollica et al., 2017). In aged mice, butyrate supplementation has been shown to induce mitochondrial biogenesis in skeletal muscles and alleviate sarcopenia (Walsh et al., 2015).

In addition, bacteria can produce metabolites that are also made by the host to influence mitochondrial functions. One example is hydrogen sulfide (H_2_S), an inhibitor of ETC complex IV component cytochrome oxidase (Blackstone et al., 2005), which can be produced in a large quantity by bacteria in the Enterobacteriaceae family (Murros, 2022). In a 15-year follow-up study, the abundance of H_2_S is associated with increased all-cause mortality (Salosensaari et al., 2021). Carbon monoxide (CO) and nitric oxide (NO) are also endogenous ETC inhibitors, and their production from the host’s microbiome promotes mitochondrial biogenesis (Hopper et al., 2020; Tengan and Moraes, 2017). Interestingly, bacteria can even modify host metabolites to regulate mitochondrial functions. In the colon, secondary bile acids (BAs) are produced by bacteria using host-derived primary BAs as substrates (Zeng et al., 2019). The secondary BA deoxycholic acid is a well-established risk factor for colon cancer (Bernstein et al., 2011), and exposure to deoxycholic acid has been reported to induce mitochondrial oxidative stress in the human colorectal carcinoma cell line HCT116 (Payne et al., 2007). Another secondary BA, tauroursodesoxycholic acid, shows neuroprotective effects in mouse models of multiple neurodegenerative diseases (Borbolis et al., 2023; Castro-Caldas et al., 2012; Keene et al., 2002; Nunes et al., 2012) and was found to promote mitophagy by upregulating mitophagy-related proteins in neuroblastoma cells (Fonseca et al., 2017). BA supplementation has been shown to extend healthspan and lifespan in a variety of organisms (Zhou et al., 2021).

**Figure 3.**
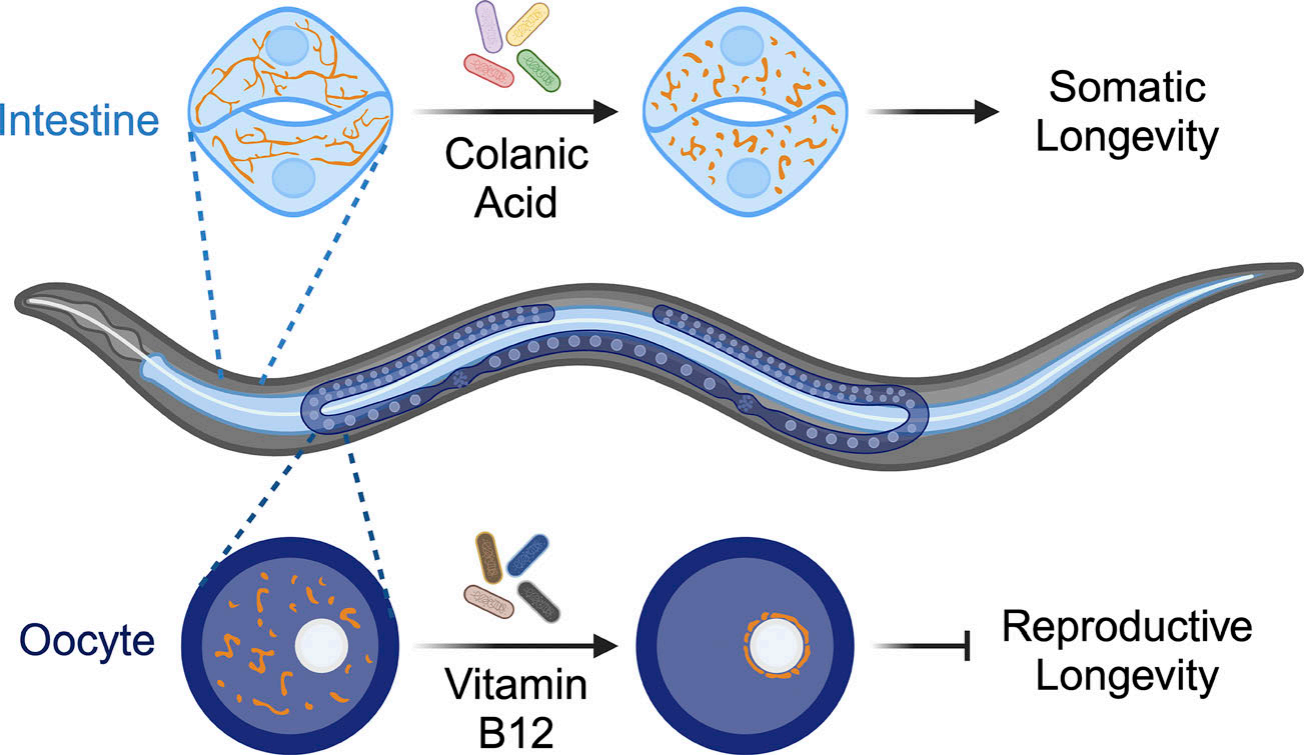
**Bacterial metabolites signal through host mitochondria to regulate somatic and reproductive aging in *C. elegans*.** Colanic acids released from bacteria direct the intestinal mitochondrial network toward fragmentation, promoting somatic longevity in *C. elegans*. Bacterial vitamin B12 causes perinuclear clustering of mitochondria in the oocyte, resulting in accelerated reproductive decline in *C. elegans*.

The model organism *C. elegans* can be supplied with a single bacterial source, facilitating the mechanistic investigation of microbe–host interactions, especially from a metabolic perspective. Through these studies, molecular mechanisms by which bacterial inputs signal through mitochondria to modulate the host’s physiology are starting to be unveiled. Colanic acid (CA) is a bacteria-secreted exopolysaccharide produced by several enterobacteriacease species, particularly *E. coli* (Grant et al., 1969). Through genome-wide screening, *E. coli* deletion strains with increased production of CA were discovered to promote longevity in the host *C. elegans* (Han et al., 2017). Interestingly, CA supplementation leads to increased mitochondrial fission in the intestine of *C. elegans* and mammalian cells (Fig. 3), and coherently, *drp-1* is required for the prolongevity effect of CA (Han et al., 2017). Other mitochondrial factors are also involved in CA-induced lifespan extension in worms. The transcription factor ATFS-1 mediates mitoUPR activation by relocating from mitochondria to the nucleus (Nargund et al., 2012). The requirement of *atfs-1* for the CA-induced prolongevity effect suggests the involvement of the mitoUPR pathway. Additionally, mitochondrial ETC components, NUO-6 of complex I and ISP-1 of complex III, both contribute to the regulation of longevity by CA (Han et al., 2017). Recent unpublished studies from our lab have revealed that this exopolysaccharide-mediated communication with mitochondria can be extended to gram-positive *Bacillus subtilis* and gram-negative *V. cholerae*.

Vitamin B12 (VB12), also known as cobalamin, varies in its levels among different bacteria with more than 1,000-fold differences in certain cases (Watson et al., 2014). B-type *E. coli* OP50 is deficient in VB12 compared with K12-type *E. coli* HT115, K12/B hybrid *E. coli* HB101, and *Comamonas aquatica* DA1877 (Lam et al., 2021; Revtovich et al., 2019; Watson et al., 2014). When exposed to VB12-deficient OP50, *C. elegans* exhibited reduced resistance to various stresses, including heat shock, pathogenic infection, and oxidative damage (Revtovich et al., 2019). Supplementation of VB12 increases the stress resistance of worms grown on OP50, which is dependent on *mmcm-1* encoding *C. elegans* methylmalonyl-CoA mutase (Revtovich et al., 2019). Methylmalonyl-CoA mutase is a VB12-dependent mitochondrial enzyme, and this dependence suggests that bacterial VB12 functions through mitochondria to influence the stress response. Furthermore, mitochondrial fragmentation increases in intestinal and muscular cells of wild-type worms grown on VB12-deficient OP50 *E. coli* (Neve et al., 2020; Revtovich et al., 2019). For the *drp-1* worm mutant, growing on VB12-rich HT115 *E. coli* leads to excess muscular mitochondrial fusion and a high rate of embryonic lethality, which can be mitigated when growing on VB12-deficient OP50 *E. coli* (Wei and Ruvkun, 2020). Interestingly, the deacidification of lysosomes, which mediate VB12 uptake, attenuates the excess mitochondrial fusion and embryonic lethality in the *drp-1* mutant grown on VB12-rich HT115 *E. coli* (Wei and Ruvkun, 2020). These results reveal that VB12 from environmental bacteria regulates mitochondrial dynamics in somatic tissues and modulates organism survival.

More recently, we discovered that bacterial VB12 regulates reproductive aging by modulating germline mitochondrial GTP levels and, subsequently, oocyte mitochondrial distribution in *C. elegans* (Fig. 3) (Lee et al., 2023). When worms grow on VB12-rich HT115 *E. coli*, the oocyte mitochondrial network is evenly dispersed throughout the cytosol at a young age but becomes clustered in the perinuclear region at an older age. This age-associated increase in perinuclear clustering is attenuated when worms grow on VB12-deficient OP50 *E. coli* but is restored with VB12 supplementation. Furthermore, worms grown on OP50 exhibit an extended reproductive lifespan, which is suppressed by VB12 supplementation. Interestingly, the age-associated perinuclear clustering of oocyte mitochondria can be also attenuated by the inactivation of GTP-specific succinyl-CoA synthetase (GTP-SCS). GTP-SCS is required for VB12 supplementation to shorten the reproductive lifespan, and germline mitochondrial GTP levels were increased with high bacterial VB12 levels during aging. Thus, bacterial VB12 can control mitochondrial GTP metabolism and mitochondrial positioning in the oocyte, consequently influencing organism reproductive health during aging.

### Bacteria–mitochondria interaction in humans

Although most mechanistic studies on the interaction between bacteria and host mitochondria are reported in model organisms, such interactions may also occur in humans and play a role in health and diseases. Polymorphisms in mitochondrial genes have been reported to be associated with the composition of the gut microbiome in humans (Ma et al., 2014). One notable observation is that mitochondrial single nucleotide polymorphisms (mtSNPs) causing synonymous mutations in key redox genes have been associated with the abundance of butyrate-producing *Clostridia* clusters IV and XIVa (Ma et al., 2014). Another study reported that a decrease in their abundance in the gut microbiome is linked to the loss of resistance against pathogen colonization (Livanos et al., 2018). This is consistent with the finding in mice, in which mtDNA polymorphism that increases mitoROS production correlates with a less diversified gut microbiome (Yardeni et al., 2019). Interestingly, aging is generally associated with increased mitoROS production (Cui et al., 2012; Giorgi et al., 2018) and decreased microbiome diversity (Aleman and Valenzano, 2019), but the causative links are still unclear.

Inflammatory bowel disease (IBD) is a collective term referring to conditions characterized by chronic inflammation of the digestive tract over an extended period, encompassing disorders such as ulcerative colitis and Crohn’s disease (CD), which have both been known to be caused by gut dysbiosis (Qiu et al., 2022). Excessive mitochondrial fragmentation (Chojnacki et al., 2023) and alterations in mitochondrial gene expression have been observed in the biopsies of patients with IBD (Haberman et al., 2019). Proteomic analysis revealed that the mitochondrial H_2_S detoxification pathway is downregulated in biopsies of CD patients (Mottawea et al., 2016). Meanwhile, the relative abundance of *Atopobium parvulum* (*A. parvulum*) positively correlates with the severity of CD in these patients, and network analysis suggests that *A. parvulum* acts as a central hub co-occurring with other known H_2_S-producing bacteria such as *Vagoccocus* and *Streptococcus* (Mottawea et al., 2016).

Gut dysbiosis is often observed in patients with neurodegenerative diseases such as PD and AD (Fang et al., 2020). IBD patients have been found to possess a higher risk of developing neurodegenerative disorders later in life, supporting that the microbiome alteration could lead to neurodegeneration (Kim et al., 2022). Microbiome analysis from stool samples suggests a reduced level of SCFAs-producing *Firmicutes* in PD patients (Hill-Burns et al., 2017; Keshavarzian et al., 2015; Mehanna et al., 2023; Petrov et al., 2017). Similarly, AD patients have a reduced level of *Firmicutes* and an elevated level of *Bacteroidetes* (Bustamante-Barrientos et al., 2023; Vogt et al., 2017), but the results are not always consistent among studies (Zhuang et al., 2018). Meanwhile, mitochondrial dysfunction and defective mitophagy are characteristics of both PD and AD (Kramer, 2021; Liu et al., 2019; Mary et al., 2023). However, how the microbiome interacts with host mitochondria to affect neurodegenerative disease progression remains inconclusive at this moment. It is possible that bacteria metabolite is involved in this process as levels of butyrate in fecal matter have been linked to PD in humans (Unger et al., 2016) as well as AD-like conditions in mice (Zhang et al., 2017). Additionally, although no direct evidence has been reported in humans, oral administration of the bacterial toxin β-N-methylamino-*L*-alanine results in neuronal mitochondrial dysfunction and fragmentation of the mitochondria network, as well as alpha-synuclein aggregation and dopaminergic neurodegeneration (Esteves et al., 2023). Gut dysbiosis resulting in gut leakiness could cause inflammatory responses of immune cells, which could be transmitted to the brain and give rise to multi-faceted neuropathology including mitochondrial dysfunction (Zhang et al., 2023). Since the presence of bacteria has been found in the brain tissue of PD and AD patients (Emery et al., 2017; Pisa et al., 2020), it is also possible that some bacteria travel to the brain to directly interact with mitochondria in the neurons.

### Bacteria interaction with other organelles

#### Pathogenic bacteria targeting chloroplasts

Chloroplasts are the site of photosynthesis in plants and some algae and are considered another significant example of endosymbiosis. It is believed that the chloroplast evolved from a photosynthetic cyanobacterium, which was engulfed by a larger eukaryotic host cell (Martin et al., 2015). The cyanobacterium survived within the host cell, and over time, the two entities developed a symbiotic relationship. The host cell provided the cyanobacterium with a protective environment to carry out photosynthesis, while the cyanobacterium provided oxygen, organic compounds, and energy to its host. Over millions of years, the cyanobacterium evolved into the chloroplast, which became an essential component of plant cells and is responsible for the production of glucose and oxygen through photosynthesis. Like mitochondria, chloroplasts are surrounded by two membranes, thought to be derived from the original engulfing of the cyanobacterium, and they also contain their own DNA. Chloroplasts are not only responsible for photosynthesis but also hold essential roles in the biosynthesis of various compounds crucial for plant growth and metabolism (Song et al., 2021). The endosymbiotic origin of chloroplasts stands as a compelling example of how symbiosis and the merging of distinct organisms can drive the evolution of complex, multicellular life forms.

Pathogenic bacteria that have evolved the ability to target chloroplasts in plant cells represent a unique facet of host-pathogen interactions (Lu and Yao, 2018). Some pathogenic bacteria release effectors that target chloroplasts, often resulting in significant alterations in host physiology and disease development. One such example is *Pseudomonas syringae* (*P. syringae*), a versatile plant pathogen with a broad host range. *P. syringae* T3SS effectors such as Hopl-1 and AvrRps4 are believed to target chloroplasts and impair photosystem II function, hampering photosynthetic electron transport (de Torres Zabala et al., 2015; Jelenska et al., 2007; Li et al., 2014). There are also examples in the *Xanthomonas* genus, which is known to infect a wide range of crops, including rice, citrus, and *Brassicaceae* vegetables. Virulence factors released from *Xanthomonas campestris* bacteria target chloroplasts, causing changes in their morphology and function, and ultimately leading to ROS accumulation in chloroplasts, photooxidative stress, and in some cases, plant death (Pierella Karlusich et al., 2017; Zurbriggen et al., 2009). The exact mechanisms by which *Xanthomonas* bacteria target and manipulate chloroplast function are not yet fully understood. In addition to *Pseudomonas* and *Xanthomonas*, another group of bacteria targeting chloroplast function is *Phytoplasms*. Once inside the plant, they infect chloroplasts, causing a range of symptoms, such as yellowing of leaves, reduced yield, dwarfing, and stunted growth (Wei et al., 2022).

#### Bacteria interaction with endosome, phagosomes, and lysosomes

Endosomes, phagosomes, and lysosomes are key cellular organelles of a dynamic network that governs the digestion of extracellular materials. Their interactions with bacteria play a vital role in pathogenic immune responses. First, phagocytosis and the formation of phagosomes are integral parts of the host’s innate immune system. When extracellular bacteria invade host cells, they can be ingested by phagocytosis and enclosed by phagosomes. The phagosomes then fuse to lysosomes to form phagolysosomes, leading to final degradation by distinct lysosomal enzymes including lysozymes (Haas, 2007). Dysfunctions in phagosomes and lysosomes can result in compromised phagocytosis and delayed bacterial degradation, and subsequently, hinder the host’s ability to combat bacterial infections (Wong et al., 2017).

Some bacteria evolve sophisticated regulatory strategies for lysosomes and phagosomes to evade lysosomal degradation (Sachdeva and Sundaramurthy, 2020). For example, *Mycobacterium tuberculosis* (Mtb) can inhibit the fusion of phagosomes with lysosomes by expressing lipoarabinomannan on their cell envelope (Gaur et al., 2014). A group of intracellular pathogenic bacteria, such as *E. coli* K1, *Leishmania donovani*, and *Salmonella enterica* also form membrane-bound vacuoles that acquire endosomal and even lysosomal membrane markers (Bakowski et al., 2010; Figueira and Holden, 2012; Garcia-del Portillo and Finlay, 1995; Kim et al., 2003; Rathman et al., 1997; Steele-Mortimer et al., 1999; Verma et al., 2017). However, these bacteria-containing vacuoles avoid the fusion with mature acidic lysosomes that carry digestive enzymes, and within the vacuole, pathogenic bacteria survive and replicate (Eswarappa et al., 2010; Kim et al., 2003; Krieger et al., 2014). These phenomena suggest that factors from these bacteria may actively inhibit endolysosomal processing and maturation. One such bacterial input identified in *E. coli* K1 is the K1 capsule, which is required for preventing the fusion of bacteria containing vacuoles and lysosomes (Kim et al., 2003). In the case of *S. enterica*, the T3SS effector SifA forms a complex with SifA and kinesin interacting protein (SKIP) and Ras-related protein 9 (RAB9) in the host cell and disrupts Rab9-mediated retrograde tracking of mannose-6-phosphate receptors, resulting in the misdirection of lysosomal enzymes and ultimately the disruption of lysosomal digestion (McGourty et al., 2012).

On the other hand, the host cell also develops adaptive responses to adjust its lysosomal homeostasis in responding to pathogenic invasion. For example, upon Mtb infection, lysosomal content and activity are greatly increased in macrophages, and this adaptive response is triggered by Mtb surface lipids sulfolipid-1 (SL-1) and mediated by mechanistic target of rapamycin complex I (mTORC1) and the transcription factor EB (TFEB) in the host (Sachdeva et al., 2020). In response to SL-1, macrophage cells also enhance the trafficking of phagosomes to lysosomes to facilitate bacterial degradation (Sachdeva et al., 2020).

#### Bacterial regulation of ER

In general, ER offers a secure environment for intracellular bacteria due to its nutrient-rich properties and limited bactericidal defenses (Celli and Tsolis, 2015). Some pathogens evolve mechanisms to manipulate the host ER to support their replication (Arasaki et al., 2012; Roy, 2002; Starr et al., 2012). For instance, *L. pneumophila*, an intravacuolar pathogen, enters host cells and resides in plasma membrane–derived vacuoles, which subsequently merge with ER-derived vesicles and form *Legionella*-containing vacuoles (Swanson and Isberg, 1995; Tilney et al., 2001). These vacuoles are enveloped by multilayer membranes adorned with ER-originated ribosomes, which are essential for bacteria to escape lysosomal digestion and replicate within the host cell (Abu Kwaik, 1996; Horwitz, 1983).

Furthermore, pathogenic bacterial infection often triggers ER stress responses, especially ER unfolded protein response (ER-UPR) (Celli and Tsolis, 2015). The secretion of virulence factors or toxins from bacteria can disrupt ER functions, leading to the accumulation of misfolded proteins (Pillich et al., 2012), and the elevated demand for cellular membrane biosynthesis places additional burdens on ER protein homeostasis (Bettigole and Glimcher, 2015). The host employs ER-UPR signaling pathways to induce immune responses, such as the production of proinflammatory cytokines (Celli and Tsolis, 2015; de Jong et al., 2013), the activation of innate immune factors (Richardson et al., 2010), and combat pathogenic infections. On the bacterial side, *Simkania negevensis*, a gram-negative intracellular bacterium, has been observed to inhibit ER-UPR, facilitating the fusion of its vacuoles with the ER and enabling its survival within host cells (Mehlitz et al., 2014).

### Summary and future perspective

The intricate interplay between bacteria and host organelles represents a multifaceted relationship of paramount importance. Examples of interactions between pathogenic bacteria and organelles illustrate how pathogens exploit these interactions to evade the host’s defenses and enhance their survival. In response, host cells leverage these connections to detect and mount defenses against pathogenic infections.

Remarkably, recent studies from our laboratory and other groups have unveiled a previously underappreciated dimension: non-pathogenic bacteria’s capacity to modulate host mitochondria (Han et al., 2017; Lee et al., 2023; Lin and Wang, 2017; Revtovich et al., 2019; Wei and Ruvkun, 2020). This modulation extends to both somatic and reproductive health, particularly in the context of aging. This revelation aligns with the understanding that mitochondria themselves have a bacterial ancestry and actively participate in the regulation of lifespan through cell non-autonomous communication between tissues (Durieux et al., 2011).

A promising future avenue of exploration lies in comprehensive and mechanistic investigations into these interactions between bacteria and mitochondria, spanning diverse host organisms. Such investigations may reveal a plethora of beneficial metabolites derived from commensal bacteria, with potential applications in promoting healthy aging, enhancing reproductive health, and preventing or treating metabolic diseases.

Furthermore, it would be equally intriguing to explore metabolite-mediated communication between commensal bacteria and other organelles, extending beyond mitochondria. A holistic exploration of these interactions promises to unravel their profound roles in host physiology and pathology. Beyond human health, the study of bacteria–organelle interactions extends to ecosystems and agriculture. Understanding how bacteria influence chloroplasts and other organelles in plants has implications for sustainable agricultural practices and environmental conservation. A deeper understanding of the intricate crosstalk between bacteria and host organelles provides a new perspective in developing approaches for promoting healthy aging and delaying age-related diseases as well as optimizing crop production and mitigating the impact of environmental stressors.

## References

[bib1] Abu Kwaik, Y. 1996. The phagosome containing *Legionella pneumophila* within the protozoan Hartmannella vermiformis is surrounded by the rough endoplasmic reticulum. Appl. Environ. Microbiol. 62:2022–2028. 10.1128/aem.62.6.2022-2028.1996 8787400 PMC167980

[bib2] Abu-Zant, A., M. Santic, M. Molmeret, S. Jones, J. Helbig, and Y. Abu Kwaik. 2005. Incomplete activation of macrophage apoptosis during intracellular replication of *Legionella pneumophila*. Infect. Immun. 73:5339–5349. 10.1128/IAI.73.9.5339-5349.2005 16113249 PMC1231138

[bib3] Abuaita, B.H., T.L. Schultz, and M.X. O’Riordan. 2018. Mitochondria-derived vesicles deliver antimicrobial reactive oxygen species to control phagosome-localized *Staphylococcus aureus*. Cell Host Microbe. 24:625–636.e5. 10.1016/j.chom.2018.10.005 30449314 PMC7323595

[bib4] Adebayo, M., S. Singh, A.P. Singh, and S. Dasgupta. 2021. Mitochondrial fusion and fission: The fine-tune balance for cellular homeostasis. FASEB J. 35:e21620. 10.1096/fj.202100067R 34048084 PMC8415099

[bib5] Aleman, F.D.D., and D.R. Valenzano. 2019. Microbiome evolution during host aging. PLoS Pathog. 15:e1007727. 10.1371/journal.ppat.1007727 31344129 PMC6657895

[bib6] Alexander, C., M. Votruba, U.E. Pesch, D.L. Thiselton, S. Mayer, A. Moore, M. Rodriguez, U. Kellner, B. Leo-Kottler, G. Auburger, . 2000. OPA1, encoding a dynamin-related GTPase, is mutated in autosomal dominant optic atrophy linked to chromosome 3q28. Nat. Genet. 26:211–215. 10.1038/79944 11017080

[bib7] Andersson, S.G., O. Karlberg, B. Canbäck, and C.G. Kurland. 2003. On the origin of mitochondria: A genomics perspective. Philos. Trans. R. Soc. Lond. B Biol. Sci. 358:165–177. 10.1098/rstb.2002.1193 12594925 PMC1693097

[bib8] Andersson, S.G., A. Zomorodipour, J.O. Andersson, T. Sicheritz-Pontén, U.C. Alsmark, R.M. Podowski, A.K. Näslund, A.S. Eriksson, H.H. Winkler, and C.G. Kurland. 1998. The genome sequence of Rickettsia prowazekii and the origin of mitochondria. Nature. 396:133–140. 10.1038/24094 9823893

[bib9] Arasaki, K., Y. Mikami, S.R. Shames, H. Inoue, Y. Wakana, and M. Tagaya. 2017. Legionella effector Lpg1137 shuts down ER-mitochondria communication through cleavage of syntaxin 17. Nat. Commun. 8:15406. 10.1038/ncomms15406 28504273 PMC5440676

[bib10] Arasaki, K., H. Shimizu, H. Mogari, N. Nishida, N. Hirota, A. Furuno, Y. Kudo, M. Baba, N. Baba, J. Cheng, . 2015. A role for the ancient SNARE syntaxin 17 in regulating mitochondrial division. Dev. Cell. 32:304–317. 10.1016/j.devcel.2014.12.011 25619926

[bib11] Arasaki, K., D.K. Toomre, and C.R. Roy. 2012. The *Legionella pneumophila* effector DrrA is sufficient to stimulate SNARE-dependent membrane fusion. Cell Host Microbe. 11:46–57. 10.1016/j.chom.2011.11.009 22264512 PMC3266541

[bib12] Arduíno, D.M., A.R. Esteves, L. Cortes, D.F. Silva, B. Patel, M. Grazina, R.H. Swerdlow, C.R. Oliveira, and S.M. Cardoso. 2012. Mitochondrial metabolism in Parkinson’s disease impairs quality control autophagy by hampering microtubule-dependent traffic. Hum. Mol. Genet. 21:4680–4702. 10.1093/hmg/dds309 22843496 PMC3471400

[bib13] Arechaga, I., and E. Cascales. 2022. Editorial: Bacterial secretion systems, Volume II. Front. Microbiol. 13:917591. 10.3389/fmicb.2022.917591 35685925 PMC9171843

[bib14] Arizmendi, O., W.D. Picking, and W.L. Picking. 2016. Macrophage apoptosis triggered by IpaD from Shigella flexneri. Infect. Immun. 84:1857–1865. 10.1128/IAI.01483-15 27068089 PMC4907132

[bib15] Arnoult, D., F. Soares, I. Tattoli, C. Castanier, D.J. Philpott, and S.E. Girardin. 2009. An N-terminal addressing sequence targets NLRX1 to the mitochondrial matrix. J. Cell Sci. 122:3161–3168. 10.1242/jcs.051193 19692591 PMC2871076

[bib16] Atteia, A., A. Adrait, S. Brugière, M. Tardif, R. van Lis, O. Deusch, T. Dagan, L. Kuhn, B. Gontero, W. Martin, . 2009. A proteomic survey of Chlamydomonas reinhardtii mitochondria sheds new light on the metabolic plasticity of the organelle and on the nature of the alpha-proteobacterial mitochondrial ancestor. Mol. Biol. Evol. 26:1533–1548. 10.1093/molbev/msp068 19349646

[bib17] Bailey, J.D., M. Diotallevi, T. Nicol, E. McNeill, A. Shaw, S. Chuaiphichai, A. Hale, A. Starr, M. Nandi, E. Stylianou, . 2019. Nitric oxide modulates metabolic remodeling in inflammatory macrophages through TCA cycle regulation and itaconate accumulation. Cell Rep. 28:218–230.e7. 10.1016/j.celrep.2019.06.018 31269442 PMC6616861

[bib18] Bakowski, M.A., V. Braun, G.Y. Lam, T. Yeung, W.D. Heo, T. Meyer, B.B. Finlay, S. Grinstein, and J.H. Brumell. 2010. The phosphoinositide phosphatase SopB manipulates membrane surface charge and trafficking of the *Salmonella*-containing vacuole. Cell Host Microbe. 7:453–462. 10.1016/j.chom.2010.05.011 20542249

[bib19] Berg, I.A., L.V. Filatova, and R.N. Ivanovsky. 2002. Inhibition of acetate and propionate assimilation by itaconate via propionyl-CoA carboxylase in isocitrate lyase-negative purple bacterium Rhodospirillum rubrum. FEMS Microbiol. Lett. 216:49–54. 10.1111/j.1574-6968.2002.tb11413.x 12423751

[bib20] Bernstein, C., H. Holubec, A.K. Bhattacharyya, H. Nguyen, C.M. Payne, B. Zaitlin, and H. Bernstein. 2011. Carcinogenicity of deoxycholate, a secondary bile acid. Arch. Toxicol. 85:863–871. 10.1007/s00204-011-0648-7 21267546 PMC3149672

[bib21] Bettigole, S.E., and L.H. Glimcher. 2015. Endoplasmic reticulum stress in immunity. Annu. Rev. Immunol. 33:107–138. 10.1146/annurev-immunol-032414-112116 25493331

[bib22] Blackstone, E., M. Morrison, and M.B. Roth. 2005. H2S induces a suspended animation-like state in mice. Science. 308:518. 10.1126/science.1108581 15845845

[bib23] Blank, M.L., J. Xia, M.M. Morcos, M. Sun, P.S. Cantrell, Y. Liu, X. Zeng, C.J. Powell, N. Yates, M.J. Boulanger, and J.P. Boyle. 2021. Toxoplasma gondii association with host mitochondria requires key mitochondrial protein import machinery. Proc. Natl. Acad. Sci. USA. 118:e2013336118. 10.1073/pnas.2013336118 PMC7999873 33723040

[bib24] Borbolis, F., E. Mytilinaiou, and K. Palikaras. 2023. The crosstalk between microbiome and mitochondrial homeostasis in neurodegeneration. Cells. 12:429. 10.3390/cells12030429 36766772 PMC9913973

[bib25] Bostick, J.W., A.M. Schonhoff, and S.K. Mazmanian. 2022. Gut microbiome-mediated regulation of neuroinflammation. Curr. Opin. Immunol. 76:102177. 10.1016/j.coi.2022.102177 35462279 PMC9167715

[bib26] Burkewitz, K., G. Feng, S. Dutta, C.A. Kelley, M. Steinbaugh, E.J. Cram, and W.B. Mair. 2020. Atf-6 regulates lifespan through ER-mitochondrial calcium homeostasis. Cell Rep. 32:108125. 10.1016/j.celrep.2020.108125 32905769 PMC8030272

[bib27] Burman, J.L., S. Pickles, C. Wang, S. Sekine, J.N.S. Vargas, Z. Zhang, A.M. Youle, C.L. Nezich, X. Wu, J.A. Hammer, and R.J. Youle. 2017. Mitochondrial fission facilitates the selective mitophagy of protein aggregates. J. Cell Biol. 216:3231–3247. 10.1083/jcb.201612106 28893839 PMC5626535

[bib28] Bustamante-Barrientos, F.A., N. Luque-Campos, M.J. Araya, E. Lara-Barba, J. de Solminihac, C. Pradenas, L. Molina, Y. Herrera-Luna, Y. Utreras-Mendoza, R. Elizondo-Vega, . 2023. Mitochondrial dysfunction in neurodegenerative disorders: Potential therapeutic application of mitochondrial transfer to central nervous system-residing cells. J. Transl. Med. 21:613. 10.1186/s12967-023-04493-w 37689642 PMC10493034

[bib29] Caruana, J.C., and S.A. Walper. 2020. Bacterial membrane vesicles as mediators of microbe - microbe and microbe - host community interactions. Front. Microbiol. 11:432. 10.3389/fmicb.2020.00432 32265873 PMC7105600

[bib30] Castro-Caldas, M., A.N. Carvalho, E. Rodrigues, C.J. Henderson, C.R. Wolf, C.M. Rodrigues, and M.J. Gama. 2012. Tauroursodeoxycholic acid prevents MPTP-induced dopaminergic cell death in a mouse model of Parkinson’s disease. Mol. Neurobiol. 46:475–486. 10.1007/s12035-012-8295-4 22773138

[bib31] Celli, J., and R.M. Tsolis. 2015. Bacteria, the endoplasmic reticulum and the unfolded protein response: Friends or foes? Nat. Rev. Microbiol. 13:71–82. 10.1038/nrmicro3393 25534809 PMC4447104

[bib32] Chan, D.C. 2006. Mitochondrial fusion and fission in mammals. Annu. Rev. Cell Dev. Biol. 22:79–99. 10.1146/annurev.cellbio.22.010305.104638 16704336

[bib33] Chaudhari, S.N., and E.T. Kipreos. 2017. Increased mitochondrial fusion allows the survival of older animals in diverse *C. elegans* longevity pathways. Nat. Commun. 8:182. 10.1038/s41467-017-00274-4 28769038 PMC5541002

[bib34] Chen, A.C., P.R. Arany, Y.Y. Huang, E.M. Tomkinson, S.K. Sharma, G.B. Kharkwal, T. Saleem, D. Mooney, F.E. Yull, T.S. Blackwell, and M.R. Hamblin. 2011. Low-level laser therapy activates NF-kB via generation of reactive oxygen species in mouse embryonic fibroblasts. PLoS One. 6:e22453. 10.1371/journal.pone.0022453 21814580 PMC3141042

[bib35] Chen, H., A. Chomyn, and D.C. Chan. 2005. Disruption of fusion results in mitochondrial heterogeneity and dysfunction. J. Biol. Chem. 280:26185–26192. 10.1074/jbc.M503062200 15899901

[bib36] Cho, I., and M.J. Blaser. 2012. The human microbiome: At the interface of health and disease. Nat. Rev. Genet. 13:260–270. 10.1038/nrg3182 22411464 PMC3418802

[bib37] Chojnacki, A.K., S. Navaneetha Krishnan, H. Jijon, T.E. Shutt, P. Colarusso, and D.M. McKay. 2023. Tissue imaging reveals disruption of epithelial mitochondrial networks and loss of mitochondria-associated cytochrome-C in inflamed human and murine colon. Mitochondrion. 68:44–59. 10.1016/j.mito.2022.10.004 36356719

[bib38] Chowdhury, S.R., A. Reimer, M. Sharan, V. Kozjak-Pavlovic, A. Eulalio, B.K. Prusty, M. Fraunholz, K. Karunakaran, and T. Rudel. 2017. Chlamydia preserves the mitochondrial network necessary for replication via microRNA-dependent inhibition of fission. J. Cell Biol. 216:1071–1089. 10.1083/jcb.201608063 28330939 PMC5379946

[bib39] Cui, H., Y. Kong, and H. Zhang. 2012. Oxidative stress, mitochondrial dysfunction, and aging. J. Signal Transduct. 2012:646354. 10.1155/2012/646354 21977319 PMC3184498

[bib40] Cullin, N., C. Azevedo Antunes, R. Straussman, C.K. Stein-Thoeringer, and E. Elinav. 2021. Microbiome and cancer. Cancer Cell. 39:1317–1341. 10.1016/j.ccell.2021.08.006 34506740

[bib41] Czajkowsky, D.M., H. Iwamoto, T.L. Cover, and Z. Shao. 1999. The vacuolating toxin from Helicobacter pylori forms hexameric pores in lipid bilayers at low pH. Proc. Natl. Acad. Sci. USA. 96:2001–2006. 10.1073/pnas.96.5.2001 10051584 PMC26726

[bib42] de Jong, M.F., T. Starr, M.G. Winter, A.B. den Hartigh, R. Child, L.A. Knodler, J.M. van Dijl, J. Celli, and R.M. Tsolis. 2013. Sensing of bacterial type IV secretion via the unfolded protein response. MBio. 4:e00418-12. 10.1128/mBio.00418-12 23422410 PMC3624511

[bib43] De Souza, D.P., A. Achuthan, M.K. Lee, K.J. Binger, M.C. Lee, S. Davidson, D.L. Tull, M.J. McConville, A.D. Cook, A.J. Murphy, . 2019. Autocrine IFN-I inhibits isocitrate dehydrogenase in the TCA cycle of LPS-stimulated macrophages. J. Clin. Invest. 129:4239–4244. 10.1172/JCI127597 31483287 PMC6763227

[bib44] de Torres Zabala, M., G. Littlejohn, S. Jayaraman, D. Studholme, T. Bailey, T. Lawson, M. Tillich, D. Licht, B. Bölter, L. Delfino, . 2015. Chloroplasts play a central role in plant defence and are targeted by pathogen effectors. Nat. Plants. 1:15074. 10.1038/nplants.2015.74 27250009

[bib45] Domańska, G., C. Motz, M. Meinecke, A. Harsman, P. Papatheodorou, B. Reljic, E.A. Dian-Lothrop, A. Galmiche, O. Kepp, L. Becker, . 2010. Helicobacter pylori VacA toxin/subunit p34: Targeting of an anion channel to the inner mitochondrial membrane. PLoS Pathog. 6:e1000878. 10.1371/journal.ppat.1000878 20442789 PMC2861713

[bib46] Durieux, J., S. Wolff, and A. Dillin. 2011. The cell-non-autonomous nature of electron transport chain-mediated longevity. Cell. 144:79–91. 10.1016/j.cell.2010.12.016 21215371 PMC3062502

[bib47] Emery, D.C., D.K. Shoemark, T.E. Batstone, C.M. Waterfall, J.A. Coghill, T.L. Cerajewska, M. Davies, N.X. West, and S.J. Allen. 2017. 16S rRNA next generation sequencing analysis shows bacteria in Alzheimer’s post-mortem brain. Front. Aging Neurosci. 9:195. 10.3389/fnagi.2017.00195 28676754 PMC5476743

[bib48] Erny, D., N. Dokalis, C. Mezö, A. Castoldi, O. Mossad, O. Staszewski, M. Frosch, M. Villa, V. Fuchs, A. Mayer, . 2021. Microbiota-derived acetate enables the metabolic fitness of the brain innate immune system during health and disease. Cell Metab. 33:2260–2276.e7. 10.1016/j.cmet.2021.10.010 34731656

[bib49] Escoll, P., M. Rolando, and C. Buchrieser. 2017a. MAMs are attractive targets for bacterial repurposing of the host cell: MAM-functions might be key for undermining an infected cell. BioEssays. 39. 10.1002/bies.201600171 28026026

[bib50] Escoll, P., O.R. Song, F. Viana, B. Steiner, T. Lagache, J.C. Olivo-Marin, F. Impens, P. Brodin, H. Hilbi, and C. Buchrieser. 2017b. Legionella pneumophila modulates mitochondrial dynamics to trigger metabolic repurposing of infected macrophages. Cell Host Microbe. 22:302–316.e7. 10.1016/j.chom.2017.07.020 28867389

[bib51] Esser, C., N. Ahmadinejad, C. Wiegand, C. Rotte, F. Sebastiani, G. Gelius-Dietrich, K. Henze, E. Kretschmann, E. Richly, D. Leister, . 2004. A genome phylogeny for mitochondria among alpha-proteobacteria and a predominantly eubacterial ancestry of yeast nuclear genes. Mol. Biol. Evol. 21:1643–1660. 10.1093/molbev/msh160 15155797

[bib52] Esteves, A.R., M.F. Munoz-Pinto, D. Nunes-Costa, E. Candeias, D.F. Silva, J.D. Magalhães, A.R. Pereira-Santos, I.L. Ferreira, S. Alarico, I. Tiago, . 2023. Footprints of a microbial toxin from the gut microbiome to mesencephalic mitochondria. Gut. 72:73–89. 10.1136/gutjnl-2021-326023 34836918 PMC9763194

[bib53] Eswarappa, S.M., V.D. Negi, S. Chakraborty, B.K. Chandrasekhar Sagar, and D. Chakravortty. 2010. Division of the *Salmonella*-containing vacuole and depletion of acidic lysosomes in *Salmonella*-infected host cells are novel strategies of *Salmonella* enterica to avoid lysosomes. Infect. Immun. 78:68–79. 10.1128/IAI.00668-09 19858305 PMC2798212

[bib54] Fan, L., D. Wu, V. Goremykin, J. Xiao, Y. Xu, S. Garg, C. Zhang, W.F. Martin, and R. Zhu. 2020. Phylogenetic analyses with systematic taxon sampling show that mitochondria branch within Alphaproteobacteria. Nat. Ecol. Evol. 4:1213–1219. 10.1038/s41559-020-1239-x 32661403

[bib55] Fang, P., S.A. Kazmi, K.G. Jameson, and E.Y. Hsiao. 2020. The microbiome as a modifier of neurodegenerative disease risk. Cell Host Microbe. 28:201–222. 10.1016/j.chom.2020.06.008 32791113 PMC7430034

[bib56] Figueira, R., and D.W. Holden. 2012. Functions of the *Salmonella* pathogenicity island 2 (SPI-2) type III secretion system effectors. Microbiology. 158:1147–1161. 10.1099/mic.0.058115-0 22422755

[bib57] Fitzpatrick, D.A., C.J. Creevey, and J.O. McInerney. 2006. Genome phylogenies indicate a meaningful alpha-proteobacterial phylogeny and support a grouping of the mitochondria with the Rickettsiales. Mol. Biol. Evol. 23:74–85. 10.1093/molbev/msj009 16151187

[bib58] Flannery, P.J., and E. Trushina. 2019. Mitochondrial dynamics and transport in Alzheimer’s disease. Mol. Cell. Neurosci. 98:109–120. 10.1016/j.mcn.2019.06.009 31216425 PMC6614006

[bib59] Flemming, H.C., J. Wingender, U. Szewzyk, P. Steinberg, S.A. Rice, and S. Kjelleberg. 2016. Biofilms: An emergent form of bacterial life. Nat. Rev. Microbiol. 14:563–575. 10.1038/nrmicro.2016.94 27510863

[bib60] Flemming, H.C., and S. Wuertz. 2019. Bacteria and archaea on Earth and their abundance in biofilms. Nat. Rev. Microbiol. 17:247–260. 10.1038/s41579-019-0158-9 30760902

[bib61] Fonseca, I., G. Gordino, S. Moreira, M.J. Nunes, C. Azevedo, M.J. Gama, E. Rodrigues, C.M.P. Rodrigues, and M. Castro-Caldas. 2017. Tauroursodeoxycholic acid protects against mitochondrial dysfunction and cell death via mitophagy in human neuroblastoma cells. Mol. Neurobiol. 54:6107–6119. 10.1007/s12035-016-0145-3 27699602

[bib62] Galmiche, A., and J. Rassow. 2010. Targeting of Helicobacter pylori VacA to mitochondria. Gut Microbes. 1:392–395. 10.4161/gmic.1.6.13894 21468222 PMC3056105

[bib63] Galmiche, A., J. Rassow, A. Doye, S. Cagnol, J.C. Chambard, S. Contamin, V. de Thillot, I. Just, V. Ricci, E. Solcia, . 2000. The N-terminal 34 kDa fragment of Helicobacter pylori vacuolating cytotoxin targets mitochondria and induces cytochrome c release. EMBO J. 19:6361–6370. 10.1093/emboj/19.23.6361 11101509 PMC305856

[bib64] Gao, Z., J. Yin, J. Zhang, R.E. Ward, R.J. Martin, M. Lefevre, W.T. Cefalu, and J. Ye. 2009. Butyrate improves insulin sensitivity and increases energy expenditure in mice. Diabetes. 58:1509–1517. 10.2337/db08-1637 19366864 PMC2699871

[bib65] Garcia-del Portillo, F., and B.B. Finlay. 1995. Targeting of *Salmonella* typhimurium to vesicles containing lysosomal membrane glycoproteins bypasses compartments with mannose 6-phosphate receptors. J. Cell Biol. 129:81–97. 10.1083/jcb.129.1.81 7698996 PMC2120372

[bib66] Gaur, R.L., K. Ren, A. Blumenthal, S. Bhamidi, F.D. González-Nilo, M. Jackson, R.N. Zare, S. Ehrt, J.D. Ernst, and N. Banaei. 2014. LprG-mediated surface expression of lipoarabinomannan is essential for virulence of *Mycobacterium tuberculosis*. PLoS Pathog. 10:e1004376. 10.1371/journal.ppat.1004376 25232742 PMC4169494

[bib67] Geiger, O., A. Sanchez-Flores, J. Padilla-Gomez, and M. Degli Esposti. 2023. Multiple approaches of cellular metabolism define the bacterial ancestry of mitochondria. Sci. Adv. 9:eadh0066. 10.1126/sciadv.adh0066 37556552 PMC10411912

[bib68] Geng, J., X. Sun, P. Wang, S. Zhang, X. Wang, H. Wu, L. Hong, C. Xie, X. Li, H. Zhao, . 2015. Kinases Mst1 and Mst2 positively regulate phagocytic induction of reactive oxygen species and bactericidal activity. Nat. Immunol. 16:1142–1152. 10.1038/ni.3268 26414765 PMC4618176

[bib69] Giorgi, C., S. Marchi, I.C.M. Simoes, Z. Ren, G. Morciano, M. Perrone, P. Patalas-Krawczyk, S. Borchard, P. Jędrak, K. Pierzynowska, . 2018. Mitochondria and reactive oxygen species in aging and age-related diseases. Int. Rev. Cell Mol. Biol. 340:209–344. 10.1016/bs.ircmb.2018.05.006 30072092 PMC8127332

[bib70] Gloire, G., S. Legrand-Poels, and J. Piette. 2006. NF-kappaB activation by reactive oxygen species: Fifteen years later. Biochem. Pharmacol. 72:1493–1505. 10.1016/j.bcp.2006.04.011 16723122

[bib71] Grant, W.D., I.W. Sutherland, and J.F. Wilkinson. 1969. Exopolysaccharide colanic acid and its occurrence in the Enterobacteriaceae. J. Bacteriol. 100:1187–1193. 10.1128/jb.100.3.1187-1193.1969 4902806 PMC250288

[bib72] Gray, M.W., G. Burger, and B.F. Lang. 1999. Mitochondrial evolution. Science. 283:1476–1481. 10.1126/science.283.5407.1476 10066161

[bib73] Gray, M.W., and W.F. Doolittle. 1982. Has the endosymbiont hypothesis been proven? Microbiol. Rev. 46:1–42. 10.1128/mr.46.1.1-42.1982 6178009 PMC373208

[bib74] Green, E.R., and J. Mecsas. 2016. Bacterial secretion systems: An overview. Microbiol. Spectr. 4. 10.1128/microbiolspec.VMBF-0012-2015 PMC4804464 26999395

[bib75] Haas, A. 2007. The phagosome: Compartment with a license to kill. Traffic. 8:311–330. 10.1111/j.1600-0854.2006.00531.x 17274798

[bib76] Haberman, Y., R. Karns, P.J. Dexheimer, M. Schirmer, J. Somekh, I. Jurickova, T. Braun, E. Novak, L. Bauman, M.H. Collins, . 2019. Ulcerative colitis mucosal transcriptomes reveal mitochondriopathy and personalized mechanisms underlying disease severity and treatment response. Nat. Commun. 10:38. 10.1038/s41467-018-07841-3 30604764 PMC6318335

[bib77] Hamasaki, M., N. Furuta, A. Matsuda, A. Nezu, A. Yamamoto, N. Fujita, H. Oomori, T. Noda, T. Haraguchi, Y. Hiraoka, . 2013. Autophagosomes form at ER-mitochondria contact sites. Nature. 495:389–393. 10.1038/nature11910 23455425

[bib78] Han, B., P. Sivaramakrishnan, C.J. Lin, I.A.A. Neve, J. He, L.W.R. Tay, J.N. Sowa, A. Sizovs, G. Du, J. Wang, . 2017. Microbial genetic composition tunes host longevity. Cell. 169:1249–1262.e13. 10.1016/j.cell.2017.05.036 28622510 PMC5635830

[bib79] Han, S.M., H. Tsuda, Y. Yang, J. Vibbert, P. Cottee, S.J. Lee, J. Winek, C. Haueter, H.J. Bellen, and M.A. Miller. 2012. Secreted VAPB/ALS8 major sperm protein domains modulate mitochondrial localization and morphology via growth cone guidance receptors. Dev. Cell. 22:348–362. 10.1016/j.devcel.2011.12.009 22264801 PMC3298687

[bib80] Hill-Burns, E.M., J.W. Debelius, J.T. Morton, W.T. Wissemann, M.R. Lewis, Z.D. Wallen, S.D. Peddada, S.A. Factor, E. Molho, C.P. Zabetian, . 2017. Parkinson’s disease and Parkinson’s disease medications have distinct signatures of the gut microbiome. Mov. Disord. 32:739–749. 10.1002/mds.26942 28195358 PMC5469442

[bib81] Hopper, C.P., L.K. De La Cruz, K.V. Lyles, L.K. Wareham, J.A. Gilbert, Z. Eichenbaum, M. Magierowski, R.K. Poole, J. Wollborn, and B. Wang. 2020. Role of carbon monoxide in host-gut microbiome communication. Chem. Rev. 120:13273–13311. 10.1021/acs.chemrev.0c00586 33089988

[bib82] Horwitz, M.A. 1983. Formation of a novel phagosome by the Legionnaires’ disease bacterium (Legionella pneumophila) in human monocytes. J. Exp. Med. 158:1319–1331. 10.1084/jem.158.4.1319 6619736 PMC2187375

[bib83] Hughes, A.L., and D.E. Gottschling. 2012. An early age increase in vacuolar pH limits mitochondrial function and lifespan in yeast. Nature. 492:261–265. 10.1038/nature11654 23172144 PMC3521838

[bib84] Hughes, D.T., and V. Sperandio. 2008. Inter-kingdom signalling: Communication between bacteria and their hosts. Nat. Rev. Microbiol. 6:111–120. 10.1038/nrmicro1836 18197168 PMC2667375

[bib85] Jabir, M.S., L. Hopkins, N.D. Ritchie, I. Ullah, H.K. Bayes, D. Li, P. Tourlomousis, A. Lupton, D. Puleston, A.K. Simon, . 2015. Mitochondrial damage contributes to *Pseudomonas aeruginosa* activation of the inflammasome and is downregulated by autophagy. Autophagy. 11:166–182. 10.4161/15548627.2014.981915 25700738 PMC4502769

[bib86] Jacobi, D., S. Liu, K. Burkewitz, N. Kory, N.H. Knudsen, R.K. Alexander, U. Unluturk, X. Li, X. Kong, A.L. Hyde, . 2015. Hepatic Bmal1 regulates rhythmic mitochondrial dynamics and promotes metabolic fitness. Cell Metab. 22:709–720. 10.1016/j.cmet.2015.08.006 26365180 PMC4598294

[bib87] Jain, P., Z.Q. Luo, and S.R. Blanke. 2011. Helicobacter pylori vacuolating cytotoxin A (VacA) engages the mitochondrial fission machinery to induce host cell death. Proc. Natl. Acad. Sci. USA. 108:16032–16037. 10.1073/pnas.1105175108 21903925 PMC3179038

[bib88] Jelenska, J., N. Yao, B.A. Vinatzer, C.M. Wright, J.L. Brodsky, and J.T. Greenberg. 2007. A J domain virulence effector of *Pseudomonas* syringae remodels host chloroplasts and suppresses defenses. Curr. Biol. 17:499–508. 10.1016/j.cub.2007.02.028 17350264 PMC1857343

[bib89] Jie, Z., H. Xia, S.L. Zhong, Q. Feng, S. Li, S. Liang, H. Zhong, Z. Liu, Y. Gao, H. Zhao, . 2017. The gut microbiome in atherosclerotic cardiovascular disease. Nat. Commun. 8:845. 10.1038/s41467-017-00900-1 29018189 PMC5635030

[bib90] Keene, C.D., C.M. Rodrigues, T. Eich, M.S. Chhabra, C.J. Steer, and W.C. Low. 2002. Tauroursodeoxycholic acid, a bile acid, is neuroprotective in a transgenic animal model of Huntington’s disease. Proc. Natl. Acad. Sci. USA. 99:10671–10676. 10.1073/pnas.162362299 12149470 PMC125009

[bib91] Keller, L., and M.G. Surette. 2006. Communication in bacteria: An ecological and evolutionary perspective. Nat. Rev. Microbiol. 4:249–258. 10.1038/nrmicro1383 16501584

[bib92] Kenny, B., and M. Jepson. 2000. Targeting of an enteropathogenic *Escherichia coli* (EPEC) effector protein to host mitochondria. Cell. Microbiol. 2:579–590. 10.1046/j.1462-5822.2000.00082.x 11207610

[bib93] Keshavarzian, A., S.J. Green, P.A. Engen, R.M. Voigt, A. Naqib, C.B. Forsyth, E. Mutlu, and K.M. Shannon. 2015. Colonic bacterial composition in Parkinson’s disease. Mov. Disord. 30:1351–1360. 10.1002/mds.26307 26179554

[bib94] Kim, G.H., Y.C. Lee, T.J. Kim, E.R. Kim, S.N. Hong, D.K. Chang, and Y.H. Kim. 2022. Risk of neurodegenerative diseases in patients with inflammatory bowel disease: A nationwide population-based cohort study. J. Crohns Colitis. 16:436–443. 10.1093/ecco-jcc/jjab162 34499125

[bib95] Kim, K.J., S.J. Elliott, F. Di Cello, M.F. Stins, and K.S. Kim. 2003. The K1 capsule modulates trafficking of *E. coli*-containing vacuoles and enhances intracellular bacterial survival in human brain microvascular endothelial cells. Cell. Microbiol. 5:245–252. 10.1046/j.1462-5822.2003.t01-1-00271.x 12675682

[bib96] Kleele, T., T. Rey, J. Winter, S. Zaganelli, D. Mahecic, H. Perreten Lambert, F.P. Ruberto, M. Nemir, T. Wai, T. Pedrazzini, and S. Manley. 2021. Distinct fission signatures predict mitochondrial degradation or biogenesis. Nature. 593:435–439. 10.1038/s41586-021-03510-6 33953403

[bib97] Kramer, P. 2021. Mitochondria-Microbiota interaction in neurodegeneration. Front. Aging Neurosci. 13:776936. 10.3389/fnagi.2021.776936 35002678 PMC8733591

[bib98] Krieger, V., D. Liebl, Y. Zhang, R. Rajashekar, P. Chlanda, K. Giesker, D. Chikkaballi, and M. Hensel. 2014. Reorganization of the endosomal system in *Salmonella*-infected cells: The ultrastructure of *Salmonella*-induced tubular compartments. PLoS Pathog. 10:e1004374. 10.1371/journal.ppat.1004374 25254663 PMC4177991

[bib99] Kröller-Schön, S., S. Steven, S. Kossmann, A. Scholz, S. Daub, M. Oelze, N. Xia, M. Hausding, Y. Mikhed, E. Zinssius, . 2014. Molecular mechanisms of the crosstalk between mitochondria and NADPH oxidase through reactive oxygen species-studies in white blood cells and in animal models. Antioxid. Redox Signal. 20:247–266. 10.1089/ars.2012.4953 23845067 PMC3887465

[bib100] Kurihara, Y., R. Itoh, A. Shimizu, N.F. Walenna, B. Chou, K. Ishii, T. Soejima, A. Fujikane, and K. Hiromatsu. 2019. Chlamydia trachomatis targets mitochondrial dynamics to promote intracellular survival and proliferation. Cell. Microbiol. 21:e12962. 10.1111/cmi.12962 30311994

[bib101] Lam, A.B., K. Kervin, and J.E. Tanis. 2021. Vitamin B_12_ impacts amyloid beta-induced proteotoxicity by regulating the methionine/S-adenosylmethionine cycle. Cell Rep. 36:109753. 10.1016/j.celrep.2021.109753 34592146 PMC8522492

[bib102] LeBlanc, J.G., F. Chain, R. Martín, L.G. Bermúdez-Humarán, S. Courau, and P. Langella. 2017. Beneficial effects on host energy metabolism of short-chain fatty acids and vitamins produced by commensal and probiotic bacteria. Microb. Cell Fact. 16:79. 10.1186/s12934-017-0691-z 28482838 PMC5423028

[bib103] Lee, Y.T., M. Savini, T. Chen, J. Yang, Q. Zhao, L. Ding, S.M. Gao, M. Senturk, J.N. Sowa, J.D. Wang, and M.C. Wang. 2023. Mitochondrial GTP metabolism controls reproductive aging in *C. elegans*. Dev. Cell. 58:2718–2731.e7. 10.1016/j.devcel.2023.08.019 37708895 PMC10842941

[bib104] Li, G., J.E. Froehlich, C. Elowsky, J. Msanne, A.C. Ostosh, C. Zhang, T. Awada, and J.R. Alfano. 2014. Distinct *Pseudomonas* type-III effectors use a cleavable transit peptide to target chloroplasts. Plant J. 77:310–321. 10.1111/tpj.12396 24299018

[bib105] Li, X., J. Straub, T.C. Medeiros, C. Mehra, F. den Brave, E. Peker, I. Atanassov, K. Stillger, J.B. Michaelis, E. Burbridge, . 2022. Mitochondria shed their outer membrane in response to infection-induced stress. Science. 375:eabi4343. 10.1126/science.abi4343 35025629

[bib106] Lima, T., T.Y. Li, A. Mottis, and J. Auwerx. 2022. Pleiotropic effects of mitochondria in aging. Nat. Aging. 2:199–213. 10.1038/s43587-022-00191-2 37118378

[bib107] Lin, C.J., and M.C. Wang. 2017. Microbial metabolites regulate host lipid metabolism through NR5A-Hedgehog signalling. Nat. Cell Biol. 19:550–557. 10.1038/ncb3515 28436966 PMC5635834

[bib108] Liu, H., W. Bao, M. Lin, H. Niu, and Y. Rikihisa. 2012. Ehrlichia type IV secretion effector ECH0825 is translocated to mitochondria and curbs ROS and apoptosis by upregulating host MnSOD. Cell. Microbiol. 14:1037–1050. 10.1111/j.1462-5822.2012.01775.x 22348527 PMC3371182

[bib109] Liu, J., W. Liu, R. Li, and H. Yang. 2019. Mitophagy in Parkinson’s disease: From pathogenesis to treatment. Cells. 8:712. 10.3390/cells8070712 31336937 PMC6678174

[bib110] Liu, Y.J., R.L. McIntyre, G.E. Janssens, and R.H. Houtkooper. 2020. Mitochondrial fission and fusion: A dynamic role in aging and potential target for age-related disease. Mech. Ageing Dev. 186:111212. 10.1016/j.mad.2020.111212 32017944

[bib111] Livanos, A.E., E.J. Snider, S. Whittier, D.H. Chong, T.C. Wang, J.A. Abrams, and D.E. Freedberg. 2018. Rapid gastrointestinal loss of Clostridial Clusters IV and XIVa in the ICU associates with an expansion of gut pathogens. PLoS One. 13:e0200322. 10.1371/journal.pone.0200322 30067768 PMC6070193

[bib112] López-García, P., and D. Moreira. 2020. The Syntrophy hypothesis for the origin of eukaryotes revisited. Nat. Microbiol. 5:655–667. 10.1038/s41564-020-0710-4 32341569

[bib113] Lu, Y., and J. Yao. 2018. Chloroplasts at the crossroad of photosynthesis, pathogen infection and plant defense. Int. J. Mol. Sci. 19:3900. 10.3390/ijms19123900 30563149 PMC6321325

[bib114] Lum, M., and R. Morona. 2014. Dynamin-related protein Drp1 and mitochondria are important for Shigella flexneri infection. Int. J. Med. Microbiol. 304:530–541. 10.1016/j.ijmm.2014.03.006 24755420

[bib115] Ma, C., M.E. Wickham, J.A. Guttman, W. Deng, J. Walker, K.L. Madsen, K. Jacobson, W.A. Vogl, B.B. Finlay, and B.A. Vallance. 2006. Citrobacter rodentium infection causes both mitochondrial dysfunction and intestinal epithelial barrier disruption in vivo: Role of mitochondrial associated protein (Map). Cell. Microbiol. 8:1669–1686. 10.1111/j.1462-5822.2006.00741.x 16759225

[bib116] Ma, J., C. Coarfa, X. Qin, P.E. Bonnen, A. Milosavljevic, J. Versalovic, and K. Aagaard. 2014. mtDNA haplogroup and single nucleotide polymorphisms structure human microbiome communities. BMC Genomics. 15:257. 10.1186/1471-2164-15-257 24694284 PMC4234434

[bib117] Martijn, J., J. Vosseberg, L. Guy, P. Offre, and T.J.G. Ettema. 2018. Deep mitochondrial origin outside the sampled alphaproteobacteria. Nature. 557:101–105. 10.1038/s41586-018-0059-5 29695865

[bib118] Martin, W., and M. Müller. 1998. The hydrogen hypothesis for the first eukaryote. Nature. 392:37–41. 10.1038/32096 9510246

[bib119] Martin, W.F., S. Garg, and V. Zimorski. 2015. Endosymbiotic theories for eukaryote origin. Philos. Trans. R. Soc. Lond. B Biol. Sci. 370:20140330. 10.1098/rstb.2014.0330 26323761 PMC4571569

[bib120] Mary, A., F. Eysert, F. Checler, and M. Chami. 2023. Mitophagy in Alzheimer’s disease: Molecular defects and therapeutic approaches. Mol. Psychiatry. 28:202–216. 10.1038/s41380-022-01631-6 35665766 PMC9812780

[bib121] McGourty, K., T.L. Thurston, S.A. Matthews, L. Pinaud, L.J. Mota, and D.W. Holden. 2012. Salmonella inhibits retrograde trafficking of mannose-6-phosphate receptors and lysosome function. Science. 338:963–967. 10.1126/science.1227037 23162002 PMC6485626

[bib122] Mehanna, M., S. AbuRaya, S.M. Ahmed, G. Ashmawy, A. Ibrahim, and E. AbdelKhaliq. 2023. Study of the gut microbiome in Egyptian patients with Parkinson’s Disease. BMC Microbiol. 23:196. 10.1186/s12866-023-02933-7 37481569 PMC10362707

[bib123] Mehlitz, A., K. Karunakaran, J.A. Herweg, G. Krohne, S. van de Linde, E. Rieck, M. Sauer, and T. Rudel. 2014. The chlamydial organism Simkania negevensis forms ER vacuole contact sites and inhibits ER-stress. Cell. Microbiol. 16:1224–1243. 10.1111/cmi.12278 24528559

[bib124] Mollica, M.P., G. Mattace Raso, G. Cavaliere, G. Trinchese, C. De Filippo, S. Aceto, M. Prisco, C. Pirozzi, F. Di Guida, A. Lama, . 2017. Butyrate regulates liver mitochondrial function, efficiency, and dynamics in insulin-resistant obese mice. Diabetes. 66:1405–1418. 10.2337/db16-0924 28223285

[bib125] Mostowy, S., M. Bonazzi, M.A. Hamon, T.N. Tham, A. Mallet, M. Lelek, E. Gouin, C. Demangel, R. Brosch, C. Zimmer, . 2010. Entrapment of intracytosolic bacteria by septin cage-like structures. Cell Host Microbe. 8:433–444. 10.1016/j.chom.2010.10.009 21075354

[bib126] Mostowy, S., V. Sancho-Shimizu, M.A. Hamon, R. Simeone, R. Brosch, T. Johansen, and P. Cossart. 2011. p62 and NDP52 proteins target intracytosolic Shigella and Listeria to different autophagy pathways. J. Biol. Chem. 286:26987–26995. 10.1074/jbc.M111.223610 21646350 PMC3143657

[bib127] Mottawea, W., C.K. Chiang, M. Mühlbauer, A.E. Starr, J. Butcher, T. Abujamel, S.A. Deeke, A. Brandel, H. Zhou, S. Shokralla, . 2016. Altered intestinal microbiota-host mitochondria crosstalk in new onset Crohn’s disease. Nat. Commun. 7:13419. 10.1038/ncomms13419 27876802 PMC5122959

[bib128] Murros, K.E. 2022. Hydrogen sulfide produced by gut bacteria may induce Parkinson’s disease. Cells. 11:978. 10.3390/cells11060978 35326429 PMC8946538

[bib129] Nagai, T., A. Abe, and C. Sasakawa. 2005. Targeting of enteropathogenic *Escherichia coli* EspF to host mitochondria is essential for bacterial pathogenesis: Critical role of the 16th leucine residue in EspF. J. Biol. Chem. 280:2998–3011. 10.1074/jbc.M411550200 15533930

[bib130] Nandi, I., L. Aroeti, R.P. Ramachandran, E.G. Kassa, E. Zlotkin-Rivkin, and B. Aroeti. 2021. Type III secreted effectors that target mitochondria. Cell. Microbiol. 23:e13352. 10.1111/cmi.13352 33960116

[bib131] Nargund, A.M., M.W. Pellegrino, C.J. Fiorese, B.M. Baker, and C.M. Haynes. 2012. Mitochondrial import efficiency of ATFS-1 regulates mitochondrial UPR activation. Science. 337:587–590. 10.1126/science.1223560 22700657 PMC3518298

[bib132] Neve, I.A.A., J.N. Sowa, C.J. Lin, P. Sivaramakrishnan, C. Herman, Y. Ye, L. Han, and M.C. Wang. 2020. *Escherichia coli* metabolite profiling leads to the development of an RNA interference strain for *Caenorhabditis elegans*. G3. 10:189–198. 10.1534/g3.119.400741 31712257 PMC6945014

[bib133] Nolan, S.J., J.D. Romano, and I. Coppens. 2017. Host lipid droplets: An important source of lipids salvaged by the intracellular parasite *Toxoplasma gondii*. PLoS Pathog. 13:e1006362. 10.1371/journal.ppat.1006362 28570716 PMC5469497

[bib134] Nougayrède, J.P., and M.S. Donnenberg. 2004. Enteropathogenic *Escherichia coli* EspF is targeted to mitochondria and is required to initiate the mitochondrial death pathway. Cell. Microbiol. 6:1097–1111. 10.1111/j.1462-5822.2004.00421.x 15469437

[bib135] Nunes, A.F., J.D. Amaral, A.C. Lo, M.B. Fonseca, R.J. Viana, Z. Callaerts-Vegh, R. D’Hooge, and C.M. Rodrigues. 2012. TUDCA, a bile acid, attenuates amyloid precursor protein processing and amyloid-β deposition in APP/PS1 mice. Mol. Neurobiol. 45:440–454. 10.1007/s12035-012-8256-y 22438081

[bib136] O’Donoghue, E.J., and A.M. Krachler. 2016. Mechanisms of outer membrane vesicle entry into host cells. Cell. Microbiol. 18:1508–1517. 10.1111/cmi.12655 27529760 PMC5091637

[bib137] O’Toole, P.W., and I.B. Jeffery. 2015. Gut microbiota and aging. Science. 350:1214–1215. 10.1126/science.aac8469 26785481

[bib138] Pallen, M.J. 2011. Time to recognise that mitochondria are bacteria? Trends Microbiol. 19:58–64. 10.1016/j.tim.2010.11.001 21123072

[bib139] Palmieri, E.M., M. Gonzalez-Cotto, W.A. Baseler, L.C. Davies, B. Ghesquière, N. Maio, C.M. Rice, T.A. Rouault, T. Cassel, R.M. Higashi, . 2020. Nitric oxide orchestrates metabolic rewiring in M1 macrophages by targeting aconitase 2 and pyruvate dehydrogenase. Nat. Commun. 11:698. 10.1038/s41467-020-14433-7 32019928 PMC7000728

[bib140] Payne, C.M., C. Weber, C. Crowley-Skillicorn, K. Dvorak, H. Bernstein, C. Bernstein, H. Holubec, B. Dvorakova, and H. Garewal. 2007. Deoxycholate induces mitochondrial oxidative stress and activates NF-kappaB through multiple mechanisms in HCT-116 colon epithelial cells. Carcinogenesis. 28:215–222. 10.1093/carcin/bgl139 16887864

[bib141] Peace, C.G., and L.A. O’Neill. 2022. The role of itaconate in host defense and inflammation. J. Clin. Invest. 132:e148548. 10.1172/JCI148548 PMC8759771 35040439

[bib142] Pernas, L., Y. Adomako-Ankomah, A.J. Shastri, S.E. Ewald, M. Treeck, J.P. Boyle, and J.C. Boothroyd. 2014. Toxoplasma effector MAF1 mediates recruitment of host mitochondria and impacts the host response. PLoS Biol. 12:e1001845. 10.1371/journal.pbio.1001845 24781109 PMC4004538

[bib143] Pernas, L., C. Bean, J.C. Boothroyd, and L. Scorrano. 2018. Mitochondria restrict growth of the intracellular parasite *Toxoplasma gondii* by limiting its uptake of fatty acids. Cell Metab. 27:886–897.e4. 10.1016/j.cmet.2018.02.018 29617646

[bib144] Petrov, V.A., I.V. Saltykova, I.A. Zhukova, V.M. Alifirova, N.G. Zhukova, Y.B. Dorofeeva, A.V. Tyakht, B.A. Kovarsky, D.G. Alekseev, E.S. Kostryukova, . 2017. Analysis of gut microbiota in patients with Parkinson’s disease. Bull. Exp. Biol. Med. 162:734–737. 10.1007/s10517-017-3700-7 28429209

[bib145] Pierella Karlusich, J.J., M.D. Zurbriggen, F. Shahinnia, S. Sonnewald, U. Sonnewald, S.A. Hosseini, M.R. Hajirezaei, and N. Carrillo. 2017. Chloroplast redox status modulates genome-wide plant responses during the non-host interaction of tobacco with the hemibiotrophic bacterium Xanthomonas campestris pv. vesicatoria. Front. Plant Sci. 8:1158. 10.3389/fpls.2017.01158 28725231 PMC5495832

[bib146] Piewngam, P., Y. Zheng, T.H. Nguyen, S.W. Dickey, H.S. Joo, A.E. Villaruz, K.A. Glose, E.L. Fisher, R.L. Hunt, B. Li, . 2018. Pathogen elimination by probiotic Bacillus via signalling interference. Nature. 562:532–537. 10.1038/s41586-018-0616-y 30305736 PMC6202238

[bib147] Pillich, H., M. Loose, K.P. Zimmer, and T. Chakraborty. 2012. Activation of the unfolded protein response by Listeria monocytogenes. Cell. Microbiol. 14:949–964. 10.1111/j.1462-5822.2012.01769.x 22321539

[bib148] Pinegin, B., N. Vorobjeva, M. Pashenkov, and B. Chernyak. 2018. The role of mitochondrial ROS in antibacterial immunity. J. Cell. Physiol. 233:3745–3754. 10.1002/jcp.26117 28771715

[bib149] Pisa, D., R. Alonso, and L. Carrasco. 2020. Parkinson’s disease: A comprehensive analysis of fungi and bacteria in brain tissue. Int. J. Biol. Sci. 16:1135–1152. 10.7150/ijbs.42257 32174790 PMC7053320

[bib150] Qiu, P., T. Ishimoto, L. Fu, J. Zhang, Z. Zhang, and Y. Liu. 2022. The gut microbiota in inflammatory bowel disease. Front. Cell. Infect. Microbiol. 12:733992. 10.3389/fcimb.2022.733992 35273921 PMC8902753

[bib151] Ramachandran, P.V., M. Savini, A.K. Folick, K. Hu, R. Masand, B.H. Graham, and M.C. Wang. 2019. Lysosomal signaling promotes longevity by adjusting mitochondrial activity. Dev. Cell. 48:685–696.e5. 10.1016/j.devcel.2018.12.022 30713071 PMC6613828

[bib152] Ramachandran, R.P., C. Spiegel, Y. Keren, T. Danieli, N. Melamed-Book, R.R. Pal, E. Zlotkin-Rivkin, I. Rosenshine, and B. Aroeti. 2020. Mitochondrial targeting of the enteropathogenic *Escherichia coli* Map triggers calcium mobilization, ADAM10-MAP kinase signaling, and host cell apoptosis. MBio. 11:e01397-20. 10.1128/mBio.01397-20 PMC7492733 32934081

[bib153] Rambold, A.S., S. Cohen, and J. Lippincott-Schwartz. 2015. Fatty acid trafficking in starved cells: Regulation by lipid droplet lipolysis, autophagy, and mitochondrial fusion dynamics. Dev. Cell. 32:678–692. 10.1016/j.devcel.2015.01.029 25752962 PMC4375018

[bib154] Rana, A., M.P. Oliveira, A.V. Khamoui, R. Aparicio, M. Rera, H.B. Rossiter, and D.W. Walker. 2017. Promoting Drp1-mediated mitochondrial fission in midlife prolongs healthy lifespan of *Drosophila melanogaster*. Nat. Commun. 8:448. 10.1038/s41467-017-00525-4 28878259 PMC5587646

[bib155] Rana, A., M. Rera, and D.W. Walker. 2013. Parkin overexpression during aging reduces proteotoxicity, alters mitochondrial dynamics, and extends lifespan. Proc. Natl. Acad. Sci. USA. 110:8638–8643. 10.1073/pnas.1216197110 23650379 PMC3666724

[bib156] Rathman, M., L.P. Barker, and S. Falkow. 1997. The unique trafficking pattern of *Salmonella* typhimurium-containing phagosomes in murine macrophages is independent of the mechanism of bacterial entry. Infect. Immun. 65:1475–1485. 10.1128/iai.65.4.1475-1485.1997 9119490 PMC175156

[bib157] Raturi, A., and T. Simmen. 2013. Where the endoplasmic reticulum and the mitochondrion tie the knot: The mitochondria-associated membrane (MAM). Biochim. Biophys. Acta. 1833:213–224. 10.1016/j.bbamcr.2012.04.013 22575682

[bib158] Reddy, P.H. 2014. Inhibitors of mitochondrial fission as a therapeutic strategy for diseases with oxidative stress and mitochondrial dysfunction. J. Alzheimers Dis. 40:245–256. 10.3233/JAD-132060 24413616 PMC3972337

[bib159] Repp, H., Z. Pamukçi, A. Koschinski, E. Domann, A. Darji, J. Birringer, D. Brockmeier, T. Chakraborty, and F. Dreyer. 2002. Listeriolysin of Listeria monocytogenes forms Ca^2+^-permeable pores leading to intracellular Ca^2+^ oscillations. Cell. Microbiol. 4:483–491. 10.1046/j.1462-5822.2002.00207.x 12174083

[bib160] Revtovich, A.V., R. Lee, and N.V. Kirienko. 2019. Interplay between mitochondria and diet mediates pathogen and stress resistance in *Caenorhabditis elegans*. PLoS Genet. 15:e1008011. 10.1371/journal.pgen.1008011 30865620 PMC6415812

[bib161] Richardson, C.E., T. Kooistra, and D.H. Kim. 2010. An essential role for XBP-1 in host protection against immune activation in *C. elegans*. Nature. 463:1092–1095. 10.1038/nature08762 20182512 PMC2834299

[bib162] Roger, A.J., S.A. Muñoz-Gómez, and R. Kamikawa. 2017. The origin and diversification of mitochondria. Curr. Biol. 27:R1177–R1192. 10.1016/j.cub.2017.09.015 29112874

[bib163] Rosenberg, G., S. Riquelme, A. Prince, and R. Avraham. 2022. Immunometabolic crosstalk during bacterial infection. Nat. Microbiol. 7:497–507. 10.1038/s41564-022-01080-5 35365784

[bib164] Roy, C.R. 2002. Exploitation of the endoplasmic reticulum by bacterial pathogens. Trends Microbiol. 10:418–424. 10.1016/S0966-842X(02)02421-6 12217507

[bib165] Sachdeva, K., M. Goel, M. Sudhakar, M. Mehta, R. Raju, K. Raman, A. Singh, and V. Sundaramurthy. 2020. Mycobacterium tuberculosis (Mtb) lipid mediated lysosomal rewiring in infected macrophages modulates intracellular Mtb trafficking and survival. J. Biol. Chem. 295:9192–9210. 10.1074/jbc.RA120.012809 32424041 PMC7335774

[bib166] Sachdeva, K., and V. Sundaramurthy. 2020. The interplay of host lysosomes and intracellular pathogens. Front. Cell. Infect. Microbiol. 10:595502. 10.3389/fcimb.2020.595502 33330138 PMC7714789

[bib167] Sagan, L. 1967. On the origin of mitosing cells. J. Theor. Biol. 14:255–274. 10.1016/0022-5193(67)90079-3 11541392

[bib168] Salazar, N., S. Arboleya, T. Fernández-Navarro, C.G. de Los Reyes-Gavilán, S. Gonzalez, and M. Gueimonde. 2019. Age-associated changes in gut microbiota and dietary components related with the immune system in adulthood and old age: A cross-sectional study. Nutrients. 11:1765. 10.3390/nu11081765 31370376 PMC6722604

[bib169] Salosensaari, A., V. Laitinen, A.S. Havulinna, G. Meric, S. Cheng, M. Perola, L. Valsta, G. Alfthan, M. Inouye, J.D. Watrous, . 2021. Taxonomic signatures of cause-specific mortality risk in human gut microbiome. Nat. Commun. 12:2671. 10.1038/s41467-021-22962-y 33976176 PMC8113604

[bib170] Sasikaran, J., M. Ziemski, P.K. Zadora, A. Fleig, and I.A. Berg. 2014. Bacterial itaconate degradation promotes pathogenicity. Nat. Chem. Biol. 10:371–377. 10.1038/nchembio.1482 24657929

[bib171] Schwartz, R.M., and M.O. Dayhoff. 1978. Origins of prokaryotes, eukaryotes, mitochondria, and chloroplasts. Science. 199:395–403. 10.1126/science.202030 202030

[bib172] Sebastián, D., E. Sorianello, J. Segalés, A. Irazoki, V. Ruiz-Bonilla, D. Sala, E. Planet, A. Berenguer-Llergo, J.P. Muñoz, M. Sánchez-Feutrie, . 2016. Mfn2 deficiency links age-related sarcopenia and impaired autophagy to activation of an adaptive mitophagy pathway. EMBO J. 35:1677–1693. 10.15252/embj.201593084 27334614 PMC4969577

[bib173] Sender, R., S. Fuchs, and R. Milo. 2016. Revised estimates for the number of human and bacteria cells in the body. PLoS Biol. 14:e1002533. 10.1371/journal.pbio.1002533 27541692 PMC4991899

[bib174] Serapio-Palacios, A., and B.B. Finlay. 2020. Dynamics of expression, secretion and translocation of type III effectors during enteropathogenic *Escherichia coli* infection. Curr. Opin. Microbiol. 54:67–76. 10.1016/j.mib.2019.12.001 32058947

[bib175] Shames, S.R., W. Deng, J.A. Guttman, C.L. de Hoog, Y. Li, P.R. Hardwidge, H.P. Sham, B.A. Vallance, L.J. Foster, and B.B. Finlay. 2010. The pathogenic *E. coli* type III effector EspZ interacts with host CD98 and facilitates host cell prosurvival signalling. Cell. Microbiol. 12:1322–1339. 10.1111/j.1462-5822.2010.01470.x 20374249

[bib176] Sheedlo, M.J., M.D. Ohi, D.B. Lacy, and T.L. Cover. 2022. Molecular architecture of bacterial type IV secretion systems. PLoS Pathog. 18:e1010720. 10.1371/journal.ppat.1010720 35951533 PMC9371333

[bib177] Shimada, K., T.R. Crother, J. Karlin, J. Dagvadorj, N. Chiba, S. Chen, V.K. Ramanujan, A.J. Wolf, L. Vergnes, D.M. Ojcius, . 2012. Oxidized mitochondrial DNA activates the NLRP3 inflammasome during apoptosis. Immunity. 36:401–414. 10.1016/j.immuni.2012.01.009 22342844 PMC3312986

[bib178] Sinai, A.P., P. Webster, and K.A. Joiner. 1997. Association of host cell endoplasmic reticulum and mitochondria with the *Toxoplasma gondii* parasitophorous vacuole membrane: A high affinity interaction. J. Cell Sci. 110:2117–2128. 10.1242/jcs.110.17.2117 9378762

[bib179] Sirianni, A., S. Krokowski, D. Lobato-Márquez, S. Buranyi, J. Pfanzelter, D. Galea, A. Willis, S. Culley, R. Henriques, G. Larrouy-Maumus, . 2016. Mitochondria mediate septin cage assembly to promote autophagy of Shigella. EMBO Rep. 17:1029–1043. 10.15252/embr.201541832 27259462 PMC4931556

[bib180] Song, Y., L. Feng, M.A.M. Alyafei, A. Jaleel, and M. Ren. 2021. Function of chloroplasts in plant stress responses. Int. J. Mol. Sci. 22:13464. 10.3390/ijms222413464 34948261 PMC8705820

[bib181] Srivastava, S. 2017. The mitochondrial basis of aging and age-related disorders. Genes. 8:398. 10.3390/genes8120398 29257072 PMC5748716

[bib182] Starr, T., R. Child, T.D. Wehrly, B. Hansen, S. Hwang, C. López-Otin, H.W. Virgin, and J. Celli. 2012. Selective subversion of autophagy complexes facilitates completion of the Brucella intracellular cycle. Cell Host Microbe. 11:33–45. 10.1016/j.chom.2011.12.002 22264511 PMC3266535

[bib183] Stavru, F., F. Bouillaud, A. Sartori, D. Ricquier, and P. Cossart. 2011. Listeria monocytogenes transiently alters mitochondrial dynamics during infection. Proc. Natl. Acad. Sci. USA. 108:3612–3617. 10.1073/pnas.1100126108 21321208 PMC3048117

[bib184] Stavru, F., A.E. Palmer, C. Wang, R.J. Youle, and P. Cossart. 2013. Atypical mitochondrial fission upon bacterial infection. Proc. Natl. Acad. Sci. USA. 110:16003–16008. 10.1073/pnas.1315784110 24043775 PMC3791707

[bib185] Steele-Mortimer, O., S. Méresse, J.P. Gorvel, B.H. Toh, and B.B. Finlay. 1999. Biogenesis of *Salmonella* typhimurium-containing vacuoles in epithelial cells involves interactions with the early endocytic pathway. Cell. Microbiol. 1:33–49. 10.1046/j.1462-5822.1999.00003.x 11207539

[bib186] Su, B., X. Wang, L. Zheng, G. Perry, M.A. Smith, and X. Zhu. 2010. Abnormal mitochondrial dynamics and neurodegenerative diseases. Biochim. Biophys. Acta. 1802:135–142. 10.1016/j.bbadis.2009.09.013 19799998 PMC2790543

[bib187] Suen, D.F., K.L. Norris, and R.J. Youle. 2008. Mitochondrial dynamics and apoptosis. Genes Dev. 22:1577–1590. 10.1101/gad.1658508 18559474 PMC2732420

[bib188] Suzuki, M., O. Danilchanka, and J.J. Mekalanos. 2014. Vibrio cholerae T3SS effector VopE modulates mitochondrial dynamics and innate immune signaling by targeting Miro GTPases. Cell Host Microbe. 16:581–591. 10.1016/j.chom.2014.09.015 25450857 PMC4391628

[bib189] Swanson, M.S., and R.R. Isberg. 1995. Association of Legionella pneumophila with the macrophage endoplasmic reticulum. Infect. Immun. 63:3609–3620. 10.1128/iai.63.9.3609-3620.1995 7642298 PMC173501

[bib190] Tattoli, I., L.A. Carneiro, M. Jéhanno, J.G. Magalhaes, Y. Shu, D.J. Philpott, D. Arnoult, and S.E. Girardin. 2008. NLRX1 is a mitochondrial NOD-like receptor that amplifies NF-kappaB and JNK pathways by inducing reactive oxygen species production. EMBO Rep. 9:293–300. 10.1038/sj.embor.7401161 18219313 PMC2267388

[bib191] Tengan, C.H., and C.T. Moraes. 2017. NO control of mitochondrial function in normal and transformed cells. Biochim. Biophys. Acta Bioenerg. 1858:573–581. 10.1016/j.bbabio.2017.02.009 28216426 PMC5487294

[bib192] Tiku, V., M.W. Tan, and I. Dikic. 2020. Mitochondrial functions in infection and immunity. Trends Cell Biol. 30:263–275. 10.1016/j.tcb.2020.01.006 32200805 PMC7126537

[bib193] Tilney, L.G., O.S. Harb, P.S. Connelly, C.G. Robinson, and C.R. Roy. 2001. How the parasitic bacterium Legionella pneumophila modifies its phagosome and transforms it into rough ER: Implications for conversion of plasma membrane to the ER membrane. J. Cell Sci. 114:4637–4650. 10.1242/jcs.114.24.4637 11792828

[bib194] Tondera, D., S. Grandemange, A. Jourdain, M. Karbowski, Y. Mattenberger, S. Herzig, S. Da Cruz, P. Clerc, I. Raschke, C. Merkwirth, . 2009. SLP-2 is required for stress-induced mitochondrial hyperfusion. EMBO J. 28:1589–1600. 10.1038/emboj.2009.89 19360003 PMC2693158

[bib195] Toyama, E.Q., S. Herzig, J. Courchet, T.L. Lewis Jr., O.C. Losón, K. Hellberg, N.P. Young, H. Chen, F. Polleux, D.C. Chan, and R.J. Shaw. 2016. Metabolism. AMP-activated protein kinase mediates mitochondrial fission in response to energy stress. Science. 351:275–281. 10.1126/science.aab4138 26816379 PMC4852862

[bib196] Toyofuku, M. 2019. Bacterial communication through membrane vesicles. Biosci. Biotechnol. Biochem. 83:1599–1605. 10.1080/09168451.2019.1608809 31021698

[bib197] Toyofuku, M., K. Morinaga, Y. Hashimoto, J. Uhl, H. Shimamura, H. Inaba, P. Schmitt-Kopplin, L. Eberl, and N. Nomura. 2017. Membrane vesicle-mediated bacterial communication. ISME J. 11:1504–1509. 10.1038/ismej.2017.13 28282039 PMC5437348

[bib198] Trakman, G.L., S. Fehily, C. Basnayake, A.L. Hamilton, E. Russell, A. Wilson-O’Brien, and M.A. Kamm. 2022. Diet and gut microbiome in gastrointestinal disease. J. Gastroenterol. Hepatol. 37:237–245. 10.1111/jgh.15728 34716949

[bib199] Tran, S.M., and M.H. Mohajeri. 2021. The role of gut bacterial metabolites in brain development, aging and disease. Nutrients. 13:732. 10.3390/nu13030732 33669008 PMC7996516

[bib200] Trimmer, P.A., R.H. Swerdlow, J.K. Parks, P. Keeney, J.P. Bennett Jr., S.W. Miller, R.E. Davis, and W.D. Parker Jr. 2000. Abnormal mitochondrial morphology in sporadic Parkinson’s and Alzheimer’s disease cybrid cell lines. Exp. Neurol. 162:37–50. 10.1006/exnr.2000.7333 10716887

[bib201] Unger, M.M., J. Spiegel, K.U. Dillmann, D. Grundmann, H. Philippeit, J. Bürmann, K. Faßbender, A. Schwiertz, and K.H. Schäfer. 2016. Short chain fatty acids and gut microbiota differ between patients with Parkinson’s disease and age-matched controls. Parkinsonism Relat. Disord. 32:66–72. 10.1016/j.parkreldis.2016.08.019 27591074

[bib202] Van Laar, V.S., and S.B. Berman. 2009. Mitochondrial dynamics in Parkinson’s disease. Exp. Neurol. 218:247–256. 10.1016/j.expneurol.2009.03.019 19332061 PMC2752687

[bib203] Verma, J.K., R. Rastogi, and A. Mukhopadhyay. 2017. Leishmania donovani resides in modified early endosomes by upregulating Rab5a expression via the downregulation of miR-494. PLoS Pathog. 13:e1006459. 10.1371/journal.ppat.1006459 28650977 PMC5501680

[bib204] Vestby, L.K., T. Grønseth, R. Simm, and L.L. Nesse. 2020. Bacterial biofilm and its role in the pathogenesis of disease. Antibiotics. 9:59. 10.3390/antibiotics9020059 32028684 PMC7167820

[bib205] Vogt, N.M., R.L. Kerby, K.A. Dill-McFarland, S.J. Harding, A.P. Merluzzi, S.C. Johnson, C.M. Carlsson, S. Asthana, H. Zetterberg, K. Blennow, . 2017. Gut microbiome alterations in Alzheimer’s disease. Sci. Rep. 7:13537. 10.1038/s41598-017-13601-y 29051531 PMC5648830

[bib206] Wakai, T., Y. Harada, K. Miyado, and T. Kono. 2014. Mitochondrial dynamics controlled by mitofusins define organelle positioning and movement during mouse oocyte maturation. Mol. Hum. Reprod. 20:1090–1100. 10.1093/molehr/gau064 25113836

[bib207] Walsh, M.E., A. Bhattacharya, K. Sataranatarajan, R. Qaisar, L. Sloane, M.M. Rahman, M. Kinter, and H. Van Remmen. 2015. The histone deacetylase inhibitor butyrate improves metabolism and reduces muscle atrophy during aging. Aging Cell. 14:957–970. 10.1111/acel.12387 26290460 PMC4693467

[bib208] Waters, C.M., and B.L. Bassler. 2005. Quorum sensing: Cell-to-cell communication in bacteria. Annu. Rev. Cell Dev. Biol. 21:319–346. 10.1146/annurev.cellbio.21.012704.131001 16212498

[bib209] Watson, E., L.T. MacNeil, A.D. Ritter, L.S. Yilmaz, A.P. Rosebrock, A.A. Caudy, and A.J. Walhout. 2014. Interspecies systems biology uncovers metabolites affecting *C. elegans* gene expression and life history traits. Cell. 156:759–770. 10.1016/j.cell.2014.01.047 24529378 PMC4169190

[bib210] Wei, W., J. Inaba, Y. Zhao, J.D. Mowery, and R. Hammond. 2022. Phytoplasma infection blocks starch breakdown and triggers chloroplast degradation, leading to premature leaf senescence, sucrose reallocation, and spatiotemporal redistribution of phytohormones. Int. J. Mol. Sci. 23:1810. 10.3390/ijms23031810 35163732 PMC8836287

[bib211] Wei, W., and G. Ruvkun. 2020. Lysosomal activity regulates *Caenorhabditis elegans* mitochondrial dynamics through vitamin B12 metabolism. Proc. Natl. Acad. Sci. USA. 117:19970–19981. 10.1073/pnas.2008021117 32737159 PMC7443905

[bib212] Weir, H.J., P. Yao, F.K. Huynh, C.C. Escoubas, R.L. Goncalves, K. Burkewitz, R. Laboy, M.D. Hirschey, and W.B. Mair. 2017. Dietary restriction and AMPK increase lifespan via mitochondrial network and peroxisome remodeling. Cell Metab. 26:884–896.e5. 10.1016/j.cmet.2017.09.024 29107506 PMC5718936

[bib213] West, A.P., I.E. Brodsky, C. Rahner, D.K. Woo, H. Erdjument-Bromage, P. Tempst, M.C. Walsh, Y. Choi, G.S. Shadel, and S. Ghosh. 2011. TLR signalling augments macrophage bactericidal activity through mitochondrial ROS. Nature. 472:476–480. 10.1038/nature09973 21525932 PMC3460538

[bib214] Westermann, B. 2010. Mitochondrial fusion and fission in cell life and death. Nat. Rev. Mol. Cell Biol. 11:872–884. 10.1038/nrm3013 21102612

[bib215] Wilmanski, T., C. Diener, N. Rappaport, S. Patwardhan, J. Wiedrick, J. Lapidus, J.C. Earls, A. Zimmer, G. Glusman, M. Robinson, . 2021. Gut microbiome pattern reflects healthy ageing and predicts survival in humans. Nat. Metab. 3:274–286. 10.1038/s42255-021-00348-0 33619379 PMC8169080

[bib216] Wong, C.O., S. Gregory, H. Hu, Y. Chao, V.E. Sepúlveda, Y. He, D. Li-Kroeger, W.E. Goldman, H.J. Bellen, and K. Venkatachalam. 2017. Lysosomal degradation is required for sustained phagocytosis of bacteria by macrophages. Cell Host Microbe. 21:719–730.e6. 10.1016/j.chom.2017.05.002 28579255 PMC5540652

[bib217] Wu, L., and Y. Luo. 2021. Bacterial quorum-sensing systems and their role in intestinal bacteria-host crosstalk. Front. Microbiol. 12:611413. 10.3389/fmicb.2021.611413 33584614 PMC7876071

[bib218] Xia, W., P. Veeragandham, Y. Cao, Y. Xu, T.E. Rhyne, J. Qian, C.W. Hung, P. Zhao, Y. Jones, H. Gao, . 2024. Obesity causes mitochondrial fragmentation and dysfunction in white adipocytes due to RalA activation. Nat. Metab. 6:273–289. 10.1038/s42255-024-00978-0 38286821 PMC10896723

[bib219] Yang, D., Y. Oyaizu, H. Oyaizu, G.J. Olsen, and C.R. Woese. 1985. Mitochondrial origins. Proc. Natl. Acad. Sci. USA. 82:4443–4447. 10.1073/pnas.82.13.4443 3892535 PMC391117

[bib220] Yang, L., Q. Long, J. Liu, H. Tang, Y. Li, F. Bao, D. Qin, D. Pei, and X. Liu. 2015. Mitochondrial fusion provides an “initial metabolic complementation” controlled by mtDNA. Cell. Mol. Life Sci. 72:2585–2598. 10.1007/s00018-015-1863-9 25708700 PMC11113443

[bib221] Yardeni, T., C.E. Tanes, K. Bittinger, L.M. Mattei, P.M. Schaefer, L.N. Singh, G.D. Wu, D.G. Murdock, and D.C. Wallace. 2019. Host mitochondria influence gut microbiome diversity: A role for ROS. Sci. Signal. 12:eaaw3159. 10.1126/scisignal.aaw3159 31266851

[bib222] Zemirli, N., E. Morel, and D. Molino. 2018. Mitochondrial dynamics in basal and stressful conditions. Int. J. Mol. Sci. 19:564. 10.3390/ijms19020564 29438347 PMC5855786

[bib223] Zeng, H., S. Umar, B. Rust, D. Lazarova, and M. Bordonaro. 2019. Secondary bile acids and short chain fatty acids in the colon: A focus on colonic microbiome, cell proliferation, inflammation, and cancer. Int. J. Mol. Sci. 20:1214. 10.3390/ijms20051214 30862015 PMC6429521

[bib224] Zhang, L., Y. Wang, X. Xiayu, C. Shi, W. Chen, N. Song, X. Fu, R. Zhou, Y.F. Xu, L. Huang, . 2017. Altered gut microbiota in a mouse model of Alzheimer’s disease. J. Alzheimers Dis. 60:1241–1257. 10.3233/JAD-170020 29036812

[bib225] Zhang, W., M. Lin, Q. Yan, K. Budachetri, L. Hou, A. Sahni, H. Liu, N.C. Han, J. Lakritz, D. Pei, and Y. Rikihisa. 2021. An intracellular nanobody targeting T4SS effector inhibits Ehrlichia infection. Proc. Natl. Acad. Sci. USA. 118:e2024102118. 10.1073/pnas.2024102118 33903242 PMC8106314

[bib226] Zhang, W., D. Xiao, Q. Mao, and H. Xia. 2023. Role of neuroinflammation in neurodegeneration development. Signal Transduct. Target. Ther. 8:267. 10.1038/s41392-023-01486-5 37433768 PMC10336149

[bib227] Zhou, Y., G. Hu, and M.C. Wang. 2021. Host and microbiota metabolic signals in aging and longevity. Nat. Chem. Biol. 17:1027–1036. 10.1038/s41589-021-00837-z 34552221

[bib228] Zhuang, Z.Q., L.L. Shen, W.W. Li, X. Fu, F. Zeng, L. Gui, Y. Lü, M. Cai, C. Zhu, Y.L. Tan, . 2018. Gut microbiota is altered in patients with Alzheimer’s disease. J. Alzheimers Dis. 63:1337–1346. 10.3233/JAD-180176 29758946

[bib229] Ziegler, D.V., D. Vindrieux, D. Goehrig, S. Jaber, G. Collin, A. Griveau, C. Wiel, N. Bendridi, S. Djebali, V. Farfariello, . 2021. Calcium channel ITPR2 and mitochondria-ER contacts promote cellular senescence and aging. Nat. Commun. 12:720. 10.1038/s41467-021-20993-z 33526781 PMC7851384

[bib230] Zinovkin, R.A., V.P. Romaschenko, I.I. Galkin, V.V. Zakharova, O.Y. Pletjushkina, B.V. Chernyak, and E.N. Popova. 2014. Role of mitochondrial reactive oxygen species in age-related inflammatory activation of endothelium. Aging. 6:661–674. 10.18632/aging.100685 25239871 PMC4169860

[bib231] Züchner, S., I.V. Mersiyanova, M. Muglia, N. Bissar-Tadmouri, J. Rochelle, E.L. Dadali, M. Zappia, E. Nelis, A. Patitucci, J. Senderek, . 2004. Mutations in the mitochondrial GTPase mitofusin 2 cause Charcot-Marie-Tooth neuropathy type 2A. Nat. Genet. 36:449–451. 10.1038/ng1341 15064763

[bib232] Zurbriggen, M.D., N. Carrillo, V.B. Tognetti, M. Melzer, M. Peisker, B. Hause, and M.R. Hajirezaei. 2009. Chloroplast-generated reactive oxygen species play a major role in localized cell death during the non-host interaction between tobacco and Xanthomonas campestris pv. vesicatoria. Plant J. 60:962–973. 10.1111/j.1365-313X.2009.04010.x 19719480

